# Relay cross metathesis reactions of vinylphosphonates

**DOI:** 10.3762/bjoc.10.201

**Published:** 2014-08-19

**Authors:** Raj K Malla, Jeremy N Ridenour, Christopher D Spilling

**Affiliations:** 1 Department of Chemistry and Biochemistry, University of Missouri St. Louis, One University Boulevard, St. Louis, MO 63121, USA

**Keywords:** centrolobine, metathesis, organo phosphorus, relay, vinyl phosphonate

## Abstract

Dimethyl (β-substituted) vinylphosphonates do not readily undergo cross metathesis reactions with Grubbs catalyst and terminal alkenes. However, the corresponding mono- or diallyl vinylphosphonate esters undergo facile cross metathesis reactions. The improved reactivity is attributed to a relay step in the cross metathesis reaction mechanism.

## Introduction

**Scheme 1:**
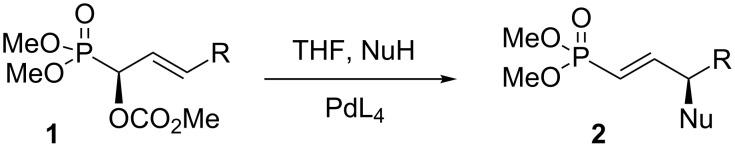
Palladium catalysed reaction of phosphono allylic carbonates.

**Figure 1:**
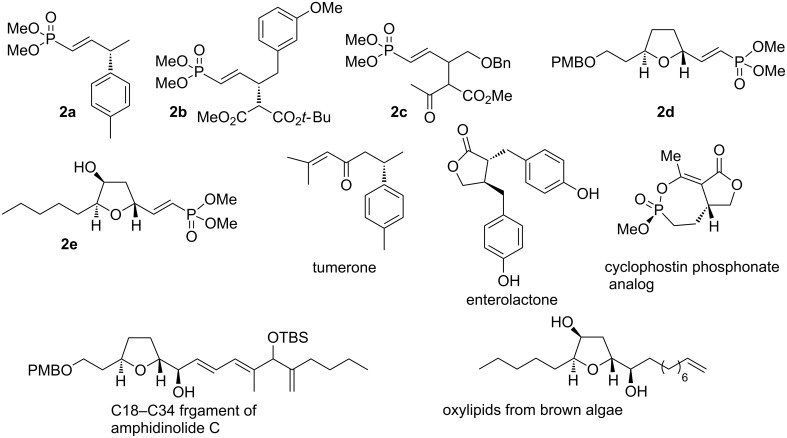
Natural products prepared using vinyl phosphonate intermediates.

Over the last two decades, we have developed reactions for the formation of chiral non-racemic γ-substituted vinylphosphonates [1–9]. In particular, carbonate derivatives **1** (phosphono allylic carbonates) of allylic hydroxy phosphonates undergo palladium-catalyzed addition of nucleophiles to give γ-substituted vinylphosphonates **2** in high yield (Scheme 1). The nucleophile adds exclusively to the 3-position, with migration of the double bond into “conjugation” with phosphoryl group. As expected, the reactions generally proceed with complete chirality transfer. Various carbon, nitrogen, and oxygen nucleophiles participate in the palladium-catalyzed substitution reactions of phosphono allylic carbonates **1**. Vinylphosphonates formed in this way, for example **2a–e** (Figure 1), have been used in the synthesis of the natural products turmerone [4] and enterolactone [5], the phosphonate derivatives of the natural product cyclophostin [6], the C18–C34 fragment of amphidinolide C [7], and the oxylipids from Australian brown algae [8].

The potential of vinylphosphonates as intermediates in organic synthesis is limited by their chemistry. Unlike the parent compound, vinylphosphonates substituted with an aryl or alkyl group on the alkene appear to have somewhat limited reactivity. This lack of reactivity is exemplified by the Grubbs cross metathesis reaction [10]. Grubbs and co-workers classified terminal vinylphosphonates as type III substrates [11]. Type III alkenes do not homodimerize, but will engage in alkene cross metathesis reactions. However, we have observed that β-substituted vinylphosphonates are unreactive towards cross metathesis and are therefore type IV substrates. Since alkene cross metathesis is a powerful method of combing organic fragments in natural product synthesis, the value of vinylphosphonates as synthetic intermediates would increase if their reactivity could be enhanced to a level where they would participate in cross metathesis reactions.

**Scheme 2:**
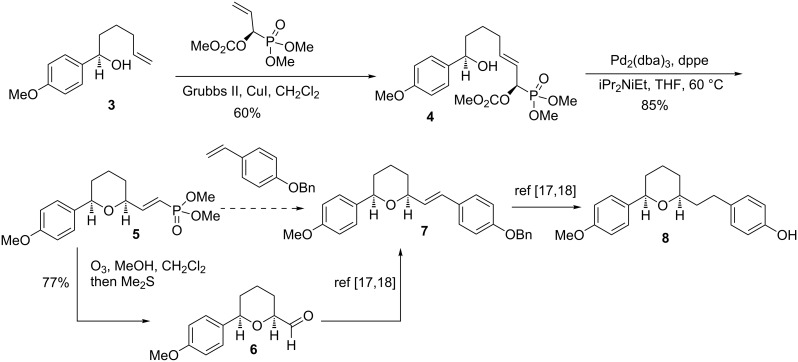
Approaches to the synthesis of centrolobine.

As an example, we recently described a method for the formal synthesis of centrolobine (**8**) [9], an antileishmananial compound isolated from the heartwood of various *Centrolobium* species [12–16] (Scheme 2)*.* The *cis*-THP substituted vinylphosphonate **5**, formed by a stereospecific palladium-catalyzed cyclization of phosphono allylic carbonate **4**, was cleaved via ozonolysis to the aldehyde **6**, a known intermediate [17–18] on route to (−)-centrolobine (**8**). An alternative approach could involve an alkene cross metathesis reaction between the vinylphosphonate and a styrene (**5** to **7**).

**Scheme 3:**
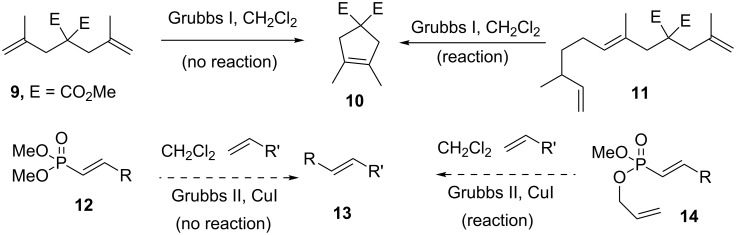
Relay ring closing metathesis and relay cross metathesis.

Since substituted vinylphosphonates are reluctant to participate in cross metathesis reactions (Scheme 3), this approach to the synthesis of cetrolobine appeared to have little merit. However, Hoye et al. reported the concept of “relay ring closing metathesis (RRCM)”, wherein typically unreactive α,ω-dienes bearing 1,1-disubstituted ethylene moieties **9** would react via the intermediacy of an additional terminal alkene **11** (Scheme 3) [19–21]. Similarly, Hansen and Lee employed an allyl ether to activate enynes toward cross metathesis [22]. Furthermore, there are several examples of vinylphosphonates participating in ring closing metathesis (RCM) reactions [23–25]. Therefore, given the propensity for vinylphosphonates to undergo RCM, it was proposed that an allyl phosphonate ester **14** would act as an initial site of metathesis, which would lead to a relay cross metathesis and thus render vinylphosphonates reactive.

## Results and Discussion

**Scheme 4:**
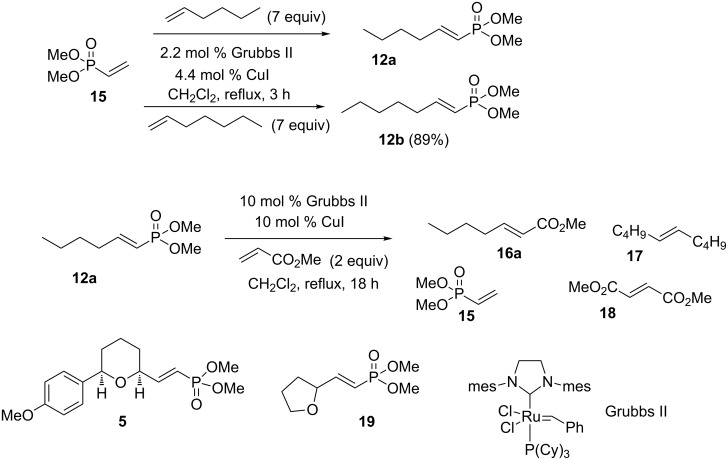
Cross metathesis reactions of vinyl phosphonates.

A series of cross metathesis reactions were performed to establish the baseline reactivity of vinylphosphonates (Scheme 4). Not surprisingly, the terminal vinylphosphonate **15** underwent smooth cross metathesis with either 1-hexene or 1-heptene using our standard conditions (2% Grubbs II, 4% CuI, CH_2_Cl_2_ reflux) [2,26–27] to give the substituted vinylphosphonates **12a** or **12b** in good yield. In contrast, when vinylphosphonate **12a** was subjected to a cross metathesis reaction with methyl acrylate, the cross metathesis product **16a** was formed in low yield (~11%) as part of a complex mixture. More highly substituted vinylphosphonates (**5** and **19**) failed to react at all with methyl acrylate under similar conditions, even with higher catalyst loading and extended reaction times.

**Scheme 5:**
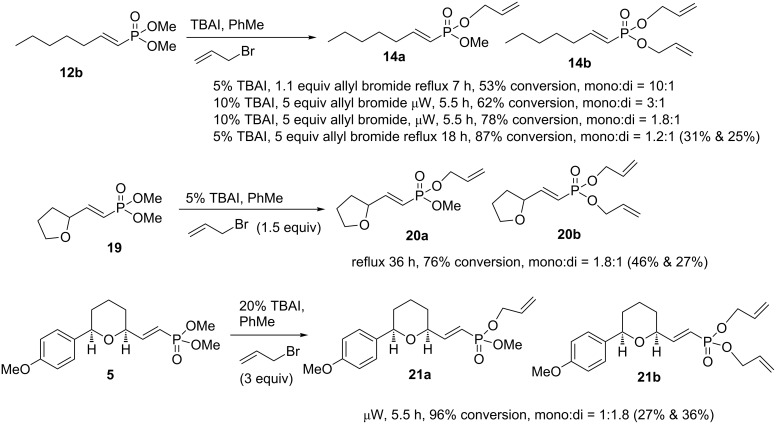
Transesterification of phosphonate esters.

Initially, the synthesis of the allyl vinylphosphonate esters was achieved using a transesterification reaction catalysed by tetra *n*-butylammonium iodide (TBAI) (Scheme 5) [28]. A solution of the vinylphosphonate **12b**, 1.1 equivalents of allyl bromide and 5 mol % TBAI in toluene was heated at reflux for 7 hours to give a 53% conversion to both the mono- and diallyl vinylphosphonates **14a** and **14b** in a 10:1 ratio. The overall conversion could be improved with excess allyl bromide, increasing the amount of TBAI and prolonged heating times, either at reflux or in a microwave reactor. The ratio of di- to mono-allyl phosphonate esters increases with the duration of reaction. A subsequent reaction of vinylphosphonate **12b** employing 5 equivalents of allyl bromide, 5 mol % TBAI and 18 hours at reflux resulted in 87% conversion with 1.2:1 ratio of mono- to diallyl ester (**14a** and **14b**). The products were isolated by silica gel chromatography to give 31% yield of mono-allyl and 25% yield of diallyl phosphonate esters.

Similarly, THF-substituted vinylphosphonate **19** was treated with 5 equivalents of allyl bromide and 5 mol % TBAI in refluxing toluene for 36 hours to give a 76% conversion with 1.8:1 ratio of mono- to diallylated vinyl phosphonates **20a** and **20b** (Scheme 5). The products were isolated by silica gel chromatography to give 46% yield of mono-allyl and 27% yield of diallyl phosphonate esters. Finally, THP-vinylphosphonate **5** was subjected to transesterification by reaction with 20 mol % of TBAI and 5 equivalents of allyl bromide in toluene solution and heating in a microwave reactor for 5.5 hours. The reaction proceeded to 96% conversion and gave 1:1.8 ratio of mono- and diallyl vinylphosphonates **21a** and **21b**. The products were isolated by silica gel chromatography to give 27% yield of the mono-allyl and 36% yield of the diallyl phosphonate esters.

**Scheme 6:**
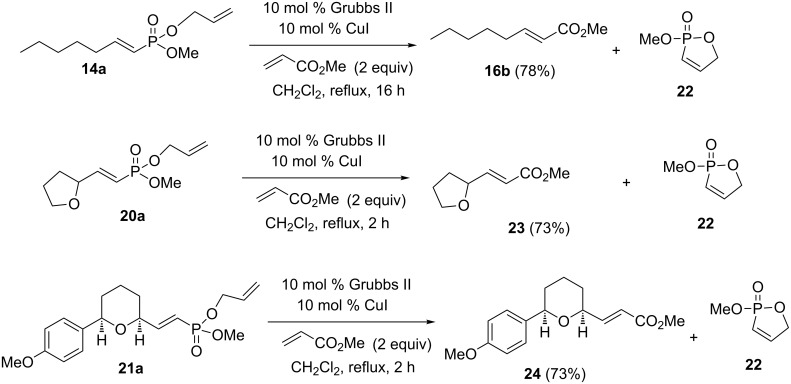
Relay cross metathesis of mono-allyl vinylphosphonates with methyl acrylate.

With the mono- and diallyl vinylphosphonates in hand, the cross metathesis reactions with methyl acrylate (a type II olefin) were examined (Scheme 6). The mono-allyl vinylphosphonate **14a** was treated with methyl acrylate, 10 mol % Grubbs catalyst and 10 mol % CuI in refluxing CH_2_Cl_2_. The unsaturated ester **16b** [29] was formed in 78% yield (estimated from NMR). However, ester **16b** is volatile and isolation by column chromatography resulted in some loss of material leading to an isolated yield of 45%. In addition, the ^31^P NMR spectrum of the crude reaction mixture indicated the formation of a new phosphorus-containing product with a signal at 43 ppm, consistent with formation of the oxaphosphole **22** [25]. An impure sample of the oxaphosphole **22** was isolated by column chromatography, but it decomposed during attempts of further purification [23]. However, the ^31^P NMR signal and the chemical shifts, multiplicities and coupling constants for the vinylic protons [Hα 7.16 (ddt, *J*_HH_ = 8.6, ~1 Hz, *J*_Hp_ = 46.9 Hz, 1H) and Hβ, 6.2 (ddt, *J*_HH_ = 8.6, 2.3 Hz, *J*_HP_ = 33.9 Hz, 1H] in the ^1^H NMR spectrum were very similar to those reported for similar structures [25] giving confidence in the structural assignment. The THF-substituted allyl vinylphosphonate **20a** and THP-substituted allyl vinylphosphonate **21a** reacted under similar conditions to yield the unsaturated esters **23** [30] and **24**, respectively.

**Scheme 7:**
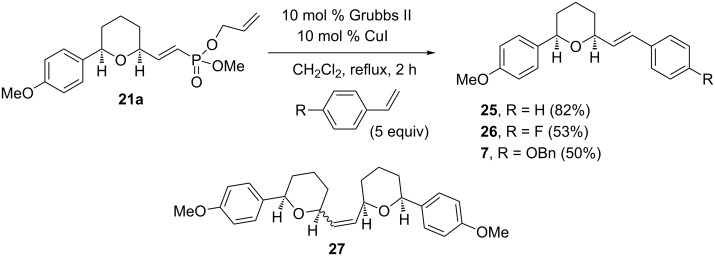
Relay cross metathesis of mono-allyl vinylphosphonates with styrenes.

The proposed synthesis of centrolobine (and analogs) (Scheme 2) required the cross metathesis reaction of the THP-substituted allyl vinylphosphonate **21a** with substituted styrenes. *p*-Substituted styrenes are type I substrates and should readily engage in the metathesis reaction. Thus, reaction of the mono-allyl vinylphosphonate **21a** with 5 equivalents of styrene using 10 mol % Grubbs second generation catalyst and 10 mol % CuI in refluxing CH_2_Cl_2_ for two hours gave tetrahydropyran **25** in 82% isolated yield (Scheme 7). Similarly, reaction of vinylphosphonate **21a** with 4-fluorostyrene and 4-benzyloxystyrene gave the tetrahydropyrans **26** and **7**, respectively. Tetrahydropyran **7** is a known intermediate and can be converted to centrolobine by hydrogenation [17].

Surprisingly, the dimer **27** was isolated in small amounts (~20%) from the reaction of vinylphosphonate **21a** with styrenes. The dimeric product **27** was not observed during the cross metathesis of the vinylphosphonate **21a** with methyl acrylate.

**Scheme 8:**
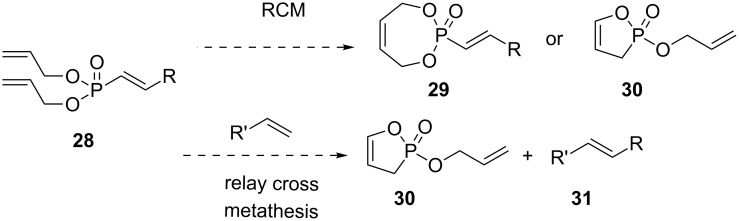
Ring closing vs relay cross metathesis.

Diallyl vinylphosphonates (**28**) are reported to undergo ring closing metathesis to give either 7-membered (**29**) or 5-membered (**30**) phosphorus heterocycles (Scheme 8) [23–24]. The mode of cyclization depends upon the geometry and substitution of the vinylphosphonate. It was proposed that (*E*) diallyl vinylphosphonates would prefer to form the 5-membered ring oxaphosphole **30** and therefore, like the corresponding mono-allyl phosphonates, should engage in relay cross metathesis reactions.

**Scheme 9:**
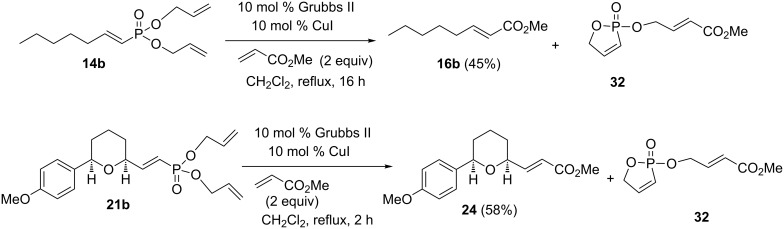
Relay cross metathesis of diallyl vinylphosphonates with methyl acrylate.

To test the hypothesis, the diallyl vinylphosphonate **14b** was subjected to cross metathesis with methyl acrylate using standard conditions (Scheme 9). The corresponding cross metathesis product, unstaurated ester **16b**, was obtained with 45% conversion. Again, isolation resulted in some loss of product. ^31^P NMR measurements also confirmed the formation of the 5-membered phosphate heterocycle **32**. Similarly, diallyl phosphonate **21b** was reacted with methyl acrylate to give the corresponding unsaturated ester **24** in good yield along with the phosphonate heterocycle **32**. In general, reaction of either mono- allyl or diallyl vinylphosphonates with methyl acrylate proceeded with comparable yields.

**Scheme 10:**
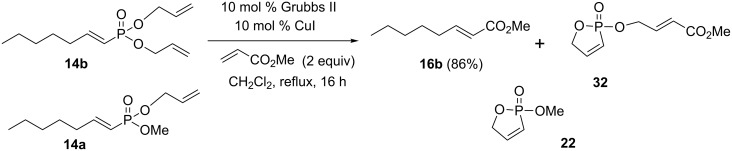
A cross metathesis reaction of both mono- and diallyl vinylphosphonates with methyl acrylate.

The mono- and diallyl vinylphosphonates were first synthesized and then chromatographically separated before they were subjected to the cross metathesis reaction. In an ideal case, a single cross metathesis product would be formed from a crude mixture of mono-allyl and diallyl vinylphosphonates, avoiding the inefficiencies of chromatographic separation. A mixture of mono- and diallyl vinylphosphonates **14a** and **14b** was subjected to cross metathesis reaction with methyl acrylate (Scheme 10). The reaction progress was monitored by ^31^P NMR spectroscopy. After the reaction was complete, the ^31^P NMR spectrum showed the formation of the two oxaphospholes **22** and **32** in a ratio corresponding to the amount of vinylphosphonates **14a** and **14b** in the starting material. Chromatographic separation of the crude product gave the unsaturated ester **16b** in 86% isolated yield.

**Scheme 11:**
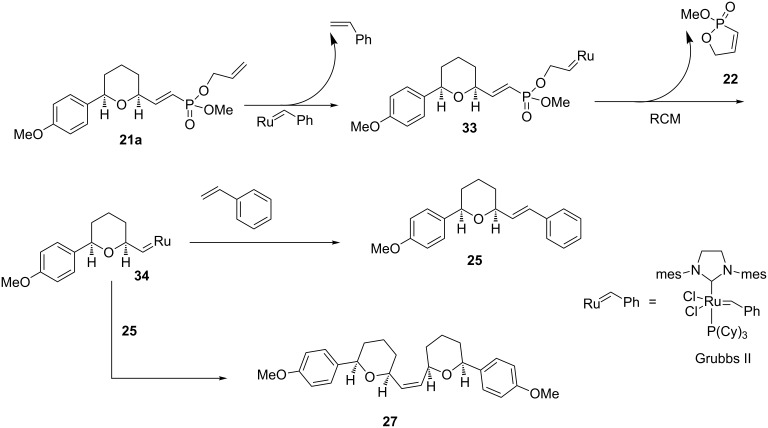
A proposed mechanism for the relay cross metathesis reaction of allyl vinylphosphonates.

It is proposed that the Grubbs catalyst first reacts with the terminal alkene (Scheme 11) of the allyl phosphonate ester **21a** to give the metal alkylidene **33**. The metal alkylidene then reacts with the vinylphosphonate in a ring closing metathesis (RCM) to generate the oxaphosphole **22** and a new metal alkylidene **34**. The sequence is completed by reaction of the metal alkylidene **34** with the metathesis partner (styrene) to give the tetrahydropyran **25**. The formation of the dimeric product **27** is probably the result of a competitive cross metathesis reaction between the tetrahydropyran **25** and the metal alkylidene **34** [31].

**Scheme 12:**
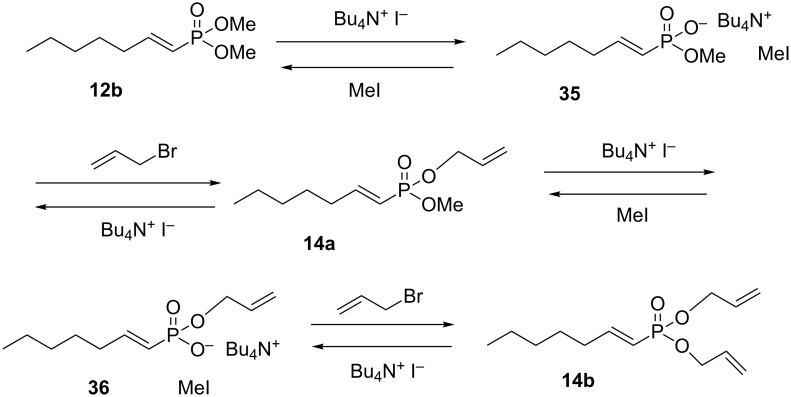
A proposed mechanism for the TBAI catalysed transesterification.

Once the activation of vinylphosphonates toward cross metathesis was established, it became clear that the overall success of this method would depend on a selective, high yielding synthesis of mono-allyl phosphonates. The proposed mechanism of the TBAI catalyzed allylation (Scheme 12) involves cleavage of the Me–O bond to form a phosphonate anion **35**. The anion is re-alkylated with allyl bromide to produce the mono-allyl phosphonate **14a**. The major weakness of this approach is that the mono-allyl phosphonate can further react with iodide leading ultimately to the diallyl phosphonate **14b**. Early in the reaction, the mono-allyl phosphonate is the dominant product. However, attempts to force the reaction with longer reaction times, increased TBAI, or increased ally bromide, leads to an increase in diallyl phosphonate **14b**.

**Scheme 13:**
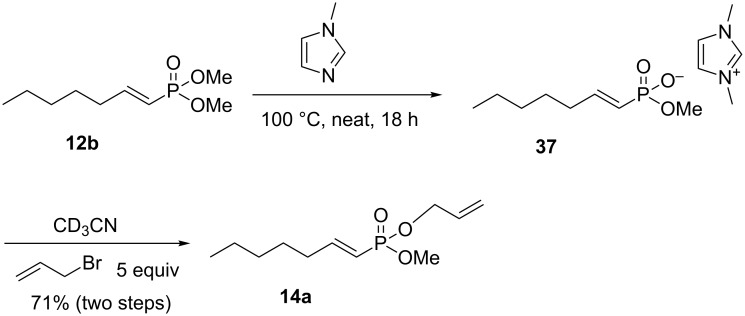
A selective synthesis of mono-allyl phosphonates.

Analysis of the TBAI allylation mechanism suggested that a good approach to mono-allyl phosphonate **14a** would be a stoichiometric demethylation followed by a rapid allylation under ambient conditions. During the synthesis of phosphonate based ionic liquids, Sachnov et al. showed that ethylimidazole would reacted with dimethyl methylphosphonate to give ethylimidazolium methylphosphonate in quantitative yield [32]. We were pleased to observe [^31^P NMR] that dimethyl vinylphosphonate **12b** reacted with neat methylimidazole at 100 °C to give the imidazolium salt **37** (Scheme 13). Treatment of the salt with 5 equivalents of allyl bromide at room temperature for two days gave the mono-allyl phosphonate in 71% isolated yield (two steps). It is probable that this transesterification reaction can be further optimized to both increase yields and decrease the reaction time.

## Conclusion

The experiments presented above have demonstrated that whereas the dimethyl esters of substituted vinylphosphonates are characterized as type IV substrates in alkene cross metathesis reactions and are unreactive, the corresponding allyl esters show significantly improved reactivity. The improved reactivity is attributed to relay step in the cross metathesis reaction mechanism.

## Supporting Information

File 1 Experimental procedures, characterization data, ^1^H and ^13^C spectra for all new compounds.

## References

[1] Shabany H, Spilling C D (1998). Tetrahedron Lett.

[2] De la Cruz A, He A, Thanavaro A, Yan B, Spilling C D, Rath N P (2005). J Organomet Chem.

[3] Yan B, Spilling C D (2008). J Org Chem.

[4] Rowe B J, Spilling C D (2003). J Org Chem.

[5] Yan B, Spilling C D (2004). J Org Chem.

[6] Bandyopadhyay S, Dutta S, Spilling C D, Dupureur C M, Rath N P (2008). J Org Chem.

[7] Roy S, Spilling C D (2010). Org Lett.

[8] Roy S, Spilling C D (2012). Org Lett.

[9] He A, Sutivisedsak N, Spilling C D (2009). Org Lett.

[10] Chatterjee A K, Choi T-L, Grubbs R H (2001). Synlett.

[11] Chatterjee A K, Choi T-L, Sanders D P, Grubbs R H (2003). J Am Chem Soc.

[12] De Albuquerque I L, Galeffi C, Casinovi C G, Marini-Bettolo G B (1964). Gazz Chim Ital.

[13] Galeffi C, Casinovi C G, Marini-Bettolo G B (1965). Gazz Chim Ital.

[14] Craveiro A A, da Costa Prado A, Gottlieb O R, Welerson de Albuquerque P C (1970). Phytochemistry.

[15] Jurd L, Wong R Y (1984). Aust J Chem.

[16] Sudarshan K, Aidhen I S (2013). Eur J Org Chem.

[17] Colobert F, Des Mazery R, Solladié G, Carreño M C (2002). Org Lett.

[18] Prasad K R, Anbarasan P (2007). Tetrahedron.

[19] Hoye T R, Jeffrey C S, Tennakoon M A, Wang J, Zhao H (2004). J Am Chem Soc.

[20] Hoye T R, Zhao H (1999). Org Lett.

[21] Hoye T R, Jeon J, Cossy J, Arseniyadis S, Meyer C (2010). Metathesis Involving a Relay and Applications in Natural Product Synthesis. Metathesis in Natural Product Synthesis: Strategies, Substrates and Catalysts.

[22] Hansen E C, Lee D (2004). Org Lett.

[23] Hanson P R, Stoianova D S (1998). Tetrahedron Lett.

[24] Hanson P R, Stoianova D R (1999). Tetrahedron Lett.

[25] Timmer M S M, Ovaa H, Filippov D V, van der Marel G A, van Boom J H (2000). Tetrahedron Lett.

[26] Rivard M, Blechert S (2003). Eur J Org Chem.

[27] He A, Yan B, Thanavaro A, Spilling C D, Rath N P (2004). J Org Chem.

[28] Malla R K, Bandyopadhyay S, Spilling C D, Dutta S, Dupureur C M (2011). Org Lett.

[29] Trost B M, Ball Z T, Jöge T (2002). J Am Chem Soc.

[30] Trost B M, Li C-J (1994). J Am Chem Soc.

[R31] 31A reviewer suggested that perhaps dimer **27** is more efficiently formed from a sequence of unproductive cross metathesis (i.e., metal exchange) of ruthenium alkylidene **34** with either ethylene or allyl phosphonate **21a**, followed by homodimerization of the resulting vinyltetrahydropyran.

[32] Sachnov S J, Schulz P S, Wasserscheid P (2011). Chem Commun.

