# Supplementary material for: Relay cross metathesis reactions of vinylphosphonates
Source: Beilstein J Org Chem. 2014 Aug 19;10:1933–41. doi: 10.3762/bjoc.10.201 (PMC4168886; doi:10.3762/bjoc.10.201)

Supporting Information

for

Relay cross metathesis reactions of vinylphosphonates

Raj K. Malla, Jeremy N. Ridenour and Christopher D. Spilling*

Address: Department of Chemistry and Biochemistry, University of Missouri St. Louis, One University Boulevard, St. Louis, MO 63121, USA

Email: Christopher D. Spilling - cspill@umsl.edu

* Corresponding author

Experimental procedures, characterization data, ^{1}H and ^{13}C spectra for all new compounds

Table of contents

General experimental	S3
Experimental procedure and spectral data for **14a** &**14b**	S3
Experimental procedure and spectral data for **20a** & **20b**	S4
Experimental procedure and spectral data for **21a** & **21b**	S5
General procedure for relay cross metathesis	S6
Experimental procedure and spectral data for **16b** & **22**	S7
Experimental procedure and spectral data for **23**	S8
Experimental procedure and spectral data for **24**	S8

Experimental procedure and spectral data for **25**	S9
Experimental procedure and spectral data for **26**	S10
Experimental procedure and spectral data for **7**	S10
Spectral data for **27**	S11
Experimental procedure for **16b** from **14b**	S11
Experimental procedure and spectral data for **24** & **32**	S12
Experimental procedure for **16b** from **14a** and **14b**	S13
Selective mono allylation of **12b**	S13–S14
^{1}H, ^{13}C NMR and ^{31}P NMR spectra of **14a**	S15–S16
^{1}H, ^{13}C NMR and ^{31}P NMR spectra of **14b**	S17–S18
^{1}H, ^{13}C NMR and ^{31}P NMR spectra of **20a**	S19–S20
^{1}H, ^{13}C NMR and ^{31}P NMR spectra of **20b**	S21–S22
^{1}H and ^{13}C NMR spectra of **21a**	S23–S24
^{1}H-^{1}H COSY and ^{31}P NMR spectrum of **21a**	S25
^{1}H, ^{13}C NMR and ^{31}P NMR spectra of **21b**	S26–S27
^{1}H-^{1}H COSY spectrum of **21b**	S28
^{1}H NMR spectrum of **16b**	S29
^{1}H NMR spectrum of **23**	S30
^{1}H and ^{13}C NMR spectra of **24**	S31–S32
^{1}H and ^{13}C NMR spectra of **25**	S33–S34
^{1}H and ^{13}C NMR spectra of **26**	S35–S36
^{1}H-^{1}H COSY spectrum of **26**	S37
^{1}H NMR and ^{1}H-^{1}H COSY spectra of **7**	S38
^{1}H-^{1}H COSY spectrum of **7**	S39
^{1}H and ^{13}C NMR spectra of **27**	S40
^{1}H-^{1}H COSY spectrum of **27**	S41
^{1}H, ^{31}P decoupled ^{1}H and ^{31}P NMR spectra of **22**	S42
^{1}H-^{1}H COSY spectrum of **22**	S43
^{1}Hand ^{13}C NMR spectra of **32**	S44
^{1}H-^{1}H COSY and ^{31}P NMR spectrum of **32**	S45
Cross metathesis of mixture of mono- and di-allyl vinylphosphonates	S46

General Experimental Details

All reactions were carried out in oven dried glassware under an atmosphere of argon unless otherwise noted. ^{1}H, ^{13}C and ^{31}P NMR spectra were recorded at 300, 75 and 121 MHz respectively. ^{1}H NMR spectra are referenced to $CDCl_3$ (7.27 ppm), ^{13}C NMR spectra are referenced to the center line of $CDCl_3$ (77.23 ppm) and ^{31}P NMR spectra are referenced to external H_3PO_4. Coupling constants, *J*, are reported in Hz.

Experimental procedure and spectral data for 14a &14b

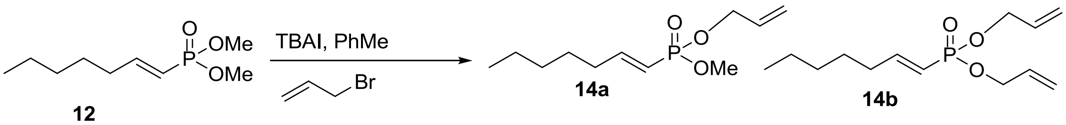

12 14a 14b

(*E*)-Allyl methyl hept-1-en-1-ylphosphonate and (*E*)-Diallyl hept-1-en-1-ylphosphonate (14a and 14b). To a solution of dimethyl vinylphosphonate **12b** (80.0 mg, 0.39 mmol) and allyl bromide (0.25 mL, 5 eq) in dry toluene (0.45 mL) was added TBAI (8 mg, 5 mol%) and the resulting mixture was heated at reflux. After 18 hours there was 87% conversion based on ^{31}P NMR analysis. The solvent was evaporated under reduced pressure and the crude product was purified by column chromatography (SiO_2, gradient 10–40 % EtOAc in hexanes) to give **14a** (28 mg, 31%) and **14b** (25 mg, 25%) as oils.

14a IR (neat, NaCl) 2958, 2929, 2859, 1633 cm^{-1}; ^{1}H NMR ($CDCl_3$) δ 6.8 (1H, ddt, J_{HH} = 17.1, 6.6 Hz, J_{HP} = 21.7 Hz), 5.93 (1H, m), 5.62 (1H, ddt, J_{HH} = 17.1, 1.6, J_{HP} = 21.7 Hz), 5.34 (1H, ddd, J_{HH} = 17.1, 3.0, 1.5 Hz), 5.23 (1H, dd, J_{HH} = 10.4, 1.3 Hz), 4.50 (2H, m), 3.70 (3H, d, J_{HP} = 11.1 Hz), 2.22 (2H, dq, J_{HH} = 7.1, 1.9 Hz), 1.44 (2H, m), 1.27 (4H, m), 0.87 (3H, t, J_{HH} = 6.8 Hz); ^{13}C NMR ($CDCl_3$) δ 155.2 (d, J_{CP} = 4.5 Hz), 133.2 (d, J_{CP} = 6.5 Hz), 118.1, 115.8 (d, J_{CP}

=187.7 Hz), 66.3 (d, J_{CP} = 5.1 Hz), 52.4 (d, J_{CP} = 5.7 Hz), 34.4 (d, J_{CP} = 22.0 Hz), 31.4, 27.6, 22.6, 14.1; ^{31}P NMR ($CDCl_3$) δ 20.8; HRMS (FAB, NBA, MH^+) calcd for $C_{11}H_{22}O_3P$: 233.1306, found 233.1305.

14b IR (neat, NaCl) 2959, 2953, 2869, 1628 cm^{-1}; ^{1}H NMR ($CDCl_3$) δ 6.81 (1H, ddt, J_{HH} = 17.1, 6.6 Hz, J_{HP} = 21.6 Hz), 5.94 (2H, m), 5.66 (1H, ddt = 17.1, 1.6 Hz, J_{HP} = 21.7 Hz), 5.34 (2H, ddd, J_{HH} = 17.1, 3.0, 1.5 Hz), 5.23 (2H, dd, J_{HH} = 10.4, 1.2 Hz), 4.51 (4H, dd, J_{HH} = 7.2, 6.1 Hz), 2.22 (2H, dq, J_{HH} = 7.1, 1.9 Hz), 1.45 (2H, m), 1.29 (4H, m), 0.88 (3H, t, J_{HH} = 6.8 Hz); ^{13}C NMR ($CDCl_3$) δ 154.9 (d, J_{CP} = 4.5 Hz), 133.2 (d, J_{CP} = 6.6 Hz), 118, 116.4 (d, J_{CP} = 187.8 Hz), 66.3 (d, J_{CP} = 5.4 Hz), 34.4 (d, J_{CP} = 22.2 Hz), 31.4, 27.6, 22.6, 14.2; ^{31}P NMR ($CDCl_3$) δ 19.8; HRMS (FAB, NBA, MH^+) calcd for $C_{13}H_{24}O_3P$: 259.1463, found 259.1455.

Experimental procedure and spectral data for 20a &20b

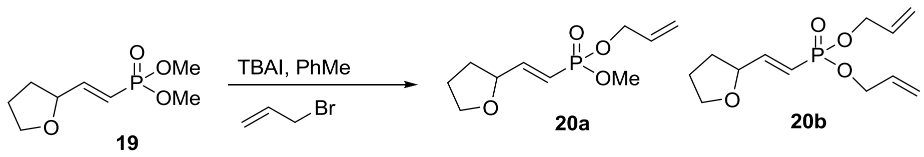

(*E*)-Allyl methyl (2-(tetrahydrofuran-2-yl)vinyl)phosphonate and (*E*)-Diallyl (2-(tetrahydrofuran-2-yl)vinyl)phosphonate (**20a** and **20b**). To a solution of vinyl phosphonate **19** (120 mg, 0.58 mmol) and allyl bromide (0.87 mL, 1.5 mmol) in dry toluene (2 mL) was added TBAI (11 mg, 5 mol%). The resulting solution was heated at reflux. After 36 hours there was 76% conversion based on ^{31}P NMR analysis. The solvent was evaporated under reduced pressure and the crude product was purified by column chromatography (SiO_2, gradient 10–40 % EtOAc in hexanes) to give **20a** (62 mg, 46%) and **20b** (40 mg, 27%) as oils.

20a IR (neat, NaCl), 2959,2955, 2865, 1635 cm^{-1}; ^{1}H NMR ($CDCl_3$) δ 6.80 (1H, ddd, J_{HH} = 17, 4.0 Hz, J_{HP} = 21 Hz), 5.93 (2H, m), 5.36 (1H, dd J_{HH} = 17.1, 1.4 Hz,), 5.25 (1H, dd, J_{HH} = 10.4,

1.4 Hz), 4.52 (3H, m), 3.92 (1H, m), 3.83 (1H, m), 3.72 (3H, d, J_{HP} = 11.2 Hz), 2.11 (1H, m), 2.94 (2H, m), 1.69 (1H, m)); ^{13}C NMR (CDCl$_3$) δ 153.9 (d, J_{CP} = 5.3 Hz), 132.3 (d, J_{CP} = 9.7 Hz), 118.1, 114.6 (d, J_{CP} =188.2 Hz), 78.5 (d, J_{CP} = 21.8 Hz), 68.8, 66.4 (d, J_{CP} = 5.3 Hz), 52.5 (d, J_{CP} = 5.9 Hz), 31.6, 25.6; ^{31}P NMR (CDCl$_3$) δ 20.75, 20.72 ppm; HRMS (FAB, MH$^+$) calcd for $C_{10}H_{18}O_4P$: 233.0943, found 233.0946.

20b IR (neat, NaCl), 2928, 2873, 1635 cm^{-1}; ^{1}H NMR (CDCl$_3$) δ 6.77 (1H, ddd, J_{HH} = 17.1, 9.5 Hz, J_{HP} = 21.5 Hz), 5.93 (3H, m), 5.34 (2H, dd J_{HH} = 17.1, 1.4 Hz), 5.22 (2H, dd, J_{HH} = 10.4, 1.3 Hz), 4.52 (5H, m), 3.93 (1H, m), 3.84 (1H, m), 2.12 (1H, m), 1.90 (2H, m), 1.66 (1H, m); ^{13}C NMR (CDCl$_3$) δ 153.6 (d, J_{CP} = 5.2 Hz), 132.2 (d, J_{CP} = 10.0 Hz), 118.1, 115.0 (d, J_{CP} =188.3 Hz), 78.4 (d, J_{CP} = 21.8 Hz), 68.7, 66.4 (d, J_{CP} = 5.3 Hz), 31.5, 25.6; ^{31}P NMR (CDCl$_3$) δ 20.4 ppm; HRMS (FAB, NBA, MH$^+$) calcd for $C_{12}H_{20}O_4P$: 259.1099, found 259.1094.

Experimental procedure and spectral data for 21a & 21b

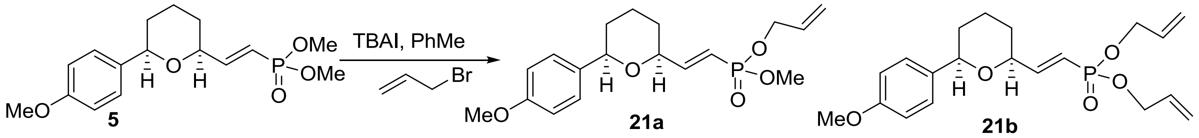

Allyl methyl ((*E*)-2-((2*S*,6*R*)-6-(4-methoxyphenyl)tetrahydro-2*H*-pyran-2-yl)vinyl) phosphonate) **and Diallyl ((*E*)-2-((2*S*,6*R*)-6-(4-methoxyphenyl)-tetrahydro-2*H*-pyran-2-yl)vinyl)phosphonate** (**21a** and **21b**)**.** To the solution of vinylphosphonate **5** (65 mg, 0.20 mmol) and allyl bromide (0.051 mL, 3 equiv.) in dry toluene (0.5 mL) was added TBAI (15 mg, 20 mol %) and the resulting mixture was heated in microwave reactor. After 5.5 hours of heating the reaction had proceeded to >96% conversion based on ^{31}P NMR analysis. The solvent was evaporated under reduced pressure and the crude product was purified by column

chromatography (SiO_2, gradient 10–40 % EtOAc in hexanes) to give pure **21a** (19 mg, 27%) and **21b** (27 mg, 36%) as oils.

21a ^{1}H NMR ($CDCl_3$) δ 7.29 (2H, d, J_{HH} = 8.6 Hz), 6.88 (2H, d, J_{HH} = 8.7 Hz), 6.80 (1H, ddd, J_{HH} = 17.1, 9.4 Hz, J_{HP} = 21.5 Hz), 6.04 (1H, dd, J_{HH} = 17.2 Hz, J_{HP} = 21.5 Hz), 5.36 (1H, dd, J_{HH} = 17.1, 1.5 Hz), 5.23 (1H, dd, J_{HH} = 10.4, 1.2 Hz), 4.52 (2H, m), 4.40 (1H, br doublet, J_{HH} = 11.2 Hz), 4.16 (1H, br doublet, J_{HH} = 11.6 Hz), 3.80 (3H, s), 3.74 (d, 1.5H, J_{HP} = 11.2 Hz), 3.72 (d, 1.5H, J_{HP} = 11.2 Hz), 1.99 (1H, m), 1.79 (2H, m), 1.69 (1H, m), 1.51 (1H, m), 1.34 (1H, m); ^{13}C NMR ($CDCl_3$) δ 159.0, 153.5 (d, J_{CP} = 5.5 Hz), 135.3, 133.1 (d, J_{CP} = 6.5 Hz), 127.1, 118.1 (d, J_{CP} = 3.3 Hz), 114.12 (d, J_{CP} = 188.3Hz), 113.8, 79.5, 66.3 (d, J_{CP} = 5.3 Hz), 55.4, 52.5 (d, J_{CP} = 5.6 Hz), 33.6, 30.9, 24.1; ^{31}P NMR ($CDCl_3$) δ 21.4, 21.3; HRMS (FAB, NBA, MH^+) calcd for $C_{18}H_{26}O_5P$: 353.1518, found 353.1524.

21b ^{1}H NMR ($CDCl_3$) δ 7.30 (2H, d, J_{HH} = 8.6 Hz), 6.89 (2H, d, J_{HH} = 8.6 Hz), 6.80 (1H, ddd, J_{HH} = 17.1, 9.4 Hz, J_{HP} = 21.5 Hz), 6.07 (1H, dd, J_{HH} = 17.1 Hz, J_{HP} = 21.5 Hz), 5.95 (2H, m), 5.35 (2H, ,m), 5.23 (2H, m), 4.52 (4H, m), 4.41 (1H, dd, J_{HH} = 10.8 Hz, J_{HP} = 1.8 Hz), 4.16 (1H, br doublet, J_{HH} = 11.6 Hz), 3.81 (3H, s), 1.99 (1H, m), 1.79 (2H, m), 1.69 (1H, m), 1.51 (1H, m), 1.34 (1H, m); ^{13}C NMR ($CDCl_3$) δ 159.0, 153.2 (d, J_{CP} = 5.5 Hz), 135.3, 133.1 (d, J_{CP} = 4.6 Hz), 127.1, 118.1 (d, J_{CP} = 3.6 Hz), 114.68 (d, J_{CP} = 188.1 Hz), 113.8, 66.3 (d, J_{CP} = 5.4 Hz), 55.4, 33.6, 30.9, 24.1; ^{31}P NMR ($CDCl_3$) δ 20.4; HRMS (FAB, NBA, MH^+) calcd for $C_{20}H_{38}O_5P$: 379.1674, found 379.1672.

General procedure for relay cross metathesis

To a solution of allyl vinylphosphonate (1 mmol) and the alkene coupling partner (2–5 mmol) in CH_2Cl_2 (2 mL) was added Grubbs 2nd generation catalyst (10 mol %) followed by CuI (10 mol %). After stirring the solution for 2 minutes at room temperature, the reaction flask was placed

in an oil bath preheated at 55 °C. After the reaction was complete (TLC and ^{31}P NMR analysis), the solvent was evaporated under reduced pressure and the crude product was purified by column chromatography (SiO_2). A gradient of 1–5% EtOAc in hexane was used to elute the cross metathesis product and then a gradient of 50–70% EtOAc in hexanes was used to elute the 5-membered phosphorus heterocycles.

Experimental procedure and spectral data for 16b & 22

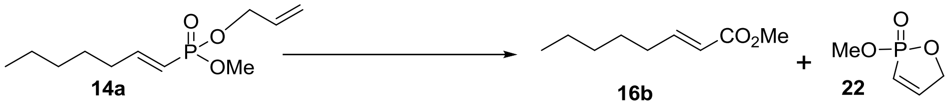

(*E*)-Methyl oct-2-enoate (16b) from reaction of mono-allyl phosphonate (14b). Allyl vinylphosphonate **14a** (54.3 mg, 0.23 mmol), methyl acrylate (42 uL, 0.46 mmol, 2 eq), Grubbs 2nd generation catalyst (20 mg, 0.023 mmol, 0.1 eq), and copper(I) iodide (4.5 mg, 0.023 mmol, 0.1 eq) were dissolved in CH_2Cl_2 (2.3 mL). The reaction flask was fitted with a reflux condenser and placed in an oil bath pre-heated to 50 °C. After 16 h, the reaction was cooled and filtered through silica gel (2 mL). The silica was rinsed with CH_2Cl_2 until just before the first dark band began eluting. The solvent was gently evaporated under reduced pressure without heating to give of crude product (44.7 mg). The yield of (*E*)-Methyl oct-2-enoate (28.5 mg, 78%) was estimated by ^{1}H NMR spectroscopy. Silica gel chromatography (15% CH_2Cl_2 in pentane) gave the pure product **16b** (16.3 mg, 45%). ^{1}H NMR ($CDCl_3$) δ 6.98 (1H, dt, J_{HH} = 15.7, 7.0 Hz), 6.01 (1H, d, J_{HH} = 15.7 Hz), 3.74 (3H, s), 2.20 (2H, m), 1.42 (2H, m), 1.28 (M, 4H), 0.91 (3H, t, J_{HH} = 7.2 Hz). The ^{1}HNMR spectral data was in agreement with the literature [1].

In other experiments, further elution gave oxaphosphole **22** in moderate purity. Any further attempt to purify the product using SiO_2 chromatography led to decomposition. **2-methoxy-2,5-dihydro-1,2-oxaphosphole 2-oxide (22).** ^{1}HNMR ($CDCl_3$) δ 7.16 (1H, ddt, J_{HH} = 8.6, ~1 Hz J_{Hp} = 46.9 Hz), 6.2 (1H, ddt, J_{HH} = 8.6, 2.3 Hz, J_{HP} = 33.9 Hz), 4.79 (2H, m), 3.75 (3H, d, J_{HP} = 11.9 Hz); ^{31}P NMR ($CDCl_3$) δ 43.7.

Experimental procedure and spectral data for 23

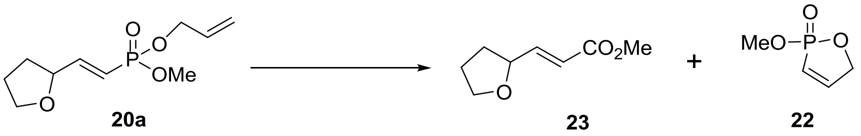

(*E*)-Methyl 3-(tetrahydrofuran-2-yl)acrylate (**23**). To a solution of allyl vinylphosphonate **20a** (9.0 mg, 0.039 mmol) and methyl acrylate (0.007 mL, 0.8 mmol) in dry CH_2Cl_2 (0.6 mL) was added Grubbs 2nd generation catalyst (3 mg, 0.004 mmol) followed by CuI (1 mg, 0.005 mmol) to give, after chromatography, the ester **23** (4 mg, 73%). ^{1}H NMR ($CDCl_3$) δ 6.91 (1H, dd, J_{HH} = 15.7, 4.8 Hz), 6.01 (1H, dd, J_{HH} = 15.7, 1.6 Hz), 4.5 (1H, dd, J_{HH} = 10.8, 2.0 Hz), 3.91 (1H, m), 3.80 (1H, m), 3.74 (3H, s), 2.11 (1H, m), 1.93 (1H, m), 1.69 (1H, m). The ^{1}HNMR spectral data was in agreement with the literature [2].

Experimental procedure and spectral data for 24

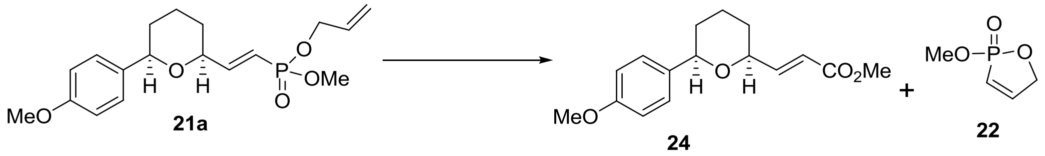

(*E*)-Methyl 3-((2*S*,6*R*)-6-(4-methoxyphenyl)tetrahydro-2*H*-pyran-2-yl)acrylate (24) from reaction of mono allyl phosphonate (21a). To a solution of allyl vinyl phosphonate **21a** (18 mg, 0.050 mmol) and methyl acrylate (0.009 mL, 0.10 mmol) in dry CH_2Cl_2 (1 mL) was added Grubbs 2nd generation catalyst (4 mg, 0.005 mmol) followed by CuI (1 mg, 0.005 mmol) to give, after chromatography, ester **24** (10 mg, 73%). ^{1}H NMR ($CDCl_3$) δ 7.30 (2H, d, J_{HH} = 8 Hz), 6.99 (1H, dd, J_{HH} = 15.7, 4.0 Hz), 6.88 (2H, d, J_{HH} = 8 Hz), 6.31 (1H, dd, J_{HH} = 15.7, 1.9 Hz), 4.41 (1H, dd, J_{HH} = 10.8, 2.0 Hz), 4.21 (1H, m), 3.81 (3H, s), 3.74 (3H, s), 2.02 (1H, m), 1.87–1.70 (3H, m), 1.59–1.50 (1H, m), 1.42–1.34 (1H, m); ^{13}C NMR ($CDCl_3$) δ 167.4, 159.0, 148.6, 135.4 127.2, 119.6, 113.8, 79.6, 55.6, 51.7, 33.6, 31.0, 24.1; HRMS (FAB, NBA/NaI, MNa$^+$) calcd for $C_{16}H_{20}O_4Na$: 299.1259, found 299.1251.

Experimental procedure and spectral data for 25

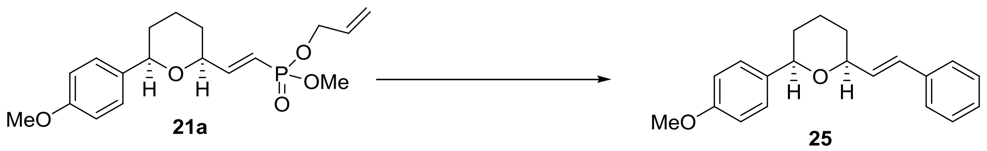

***(2R,6S)*-2-(4-Methoxyphenyl)-6-((*E*)-styryl)tetrahydro-2*H*-pyran (25).** To the solution of allyl vinylphosphonate **21a** (20 mg, 0.06 mmol) and styrene (0.013 mL, 0.11 mmol) in dry CH_2Cl_2 (1 mL) was added Grubbs 2nd generation catalyst (5 mg, 0.006 mmol) followed by CuI (1 mg, 0.006 mmol) to give, after chromatography, pure **25** (14 mg, 82%). IR (neat, NaCl) 2927, 2850, 1605 cm^{-1}; ^{1}H NMR ($CDCl_3$) δ 7.44 7.21 (7H, m), 6.92 (2H, d, J_{HH} = 8.8 Hz), 6.89 (2H, d, J_{HH} = 15.9 Hz), 6.33 (1H, dd, J_{HH} = 16.0, 5.8 Hz), 4.45 (1H, dd, J_{HH} = 11.1, 1.7 Hz), 4.21 (1H, m), 3.82 (3H, s), 2.02 (1H, m), 1.87-1.70 (3H, m), 1.66-1.50 (1H, m); ^{13}C NMR ($CDCl_3$) δ 159.1, 137.1, 135.8, 131.2, 129.9, 128.7, 127.8, 127.5, 126.7, 113.9, 79.8, 78.9, 55.5, 33.6, 31.9, 24.3; HRMS (FAB, MH$^+$) calcd for $C_{20}H_{22}O_2$: 294.1619, found 294.1618.

Experimental procedure and spectral data for 26

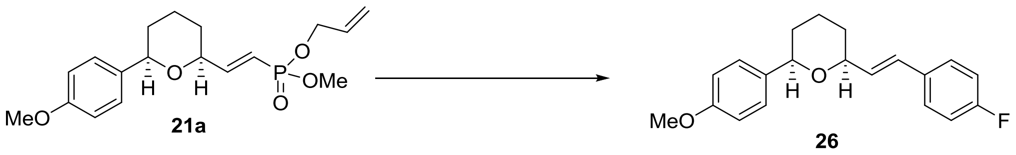

(2*S*,6*R*)-2-(4-Fluorostyryl)-6-(4-methoxyphenyl)tetrahydro-2*H*-pyran (26). To a solution of allyl vinylphosphonate **21a** (0.035 g, 0.1 mmol) and 4-fluorostyrene (0.025 mL, 0.21 mmol) in dry CH_2Cl_2 (1.6 mL) was added Grubbs 2nd generation catalyst (0.009 g, 0.01 mmol) followed by CuI (0.002 g, 0.01 mmol) to give, after chromatography, pure **26** (0.017g, 53%). ^{1}H NMR ($CDCl_3$) δ 7.36 (4H, m), 7.03 (2H, m), 6.89 (2H, m), 6.61 (1H, d, J_{HH} = 16.1 Hz), 6.22 (1H,dd, J_{HH} = 16.1, 5.8 Hz), 4.40 (1H, d, J_{HH} = 14.1, 1.9 Hz), 4.20 (1H, dd, J_{HH} = 11.0, 5.7 Hz), 3.82 (3H, s), 2.00 (1H, m), 1.82 (3H, m), 1.56 (2H, m); ^{13}C NMR ($CDCl_3$) δ 162.4 (d, J_{CF} = 244.9 Hz), 159.1, 135.7, 133.5 (d, J_{CF} = 3.2 Hz),130.9 (d, J_{CF} = 2.1 Hz), 128.7, 128.1 (d, J_{CF} = 8.0 Hz), 127.5, 115.7, 115.4, 113.9, 79.8, 78.8, 55.5, 33.6, 31.9, 24.2. HRMS (FAB, NBA, M^+) calcd for $C_{20}H_{21}O_2F$: 312.31526, found 312.1524.

Experimental procedure and spectral data for 7

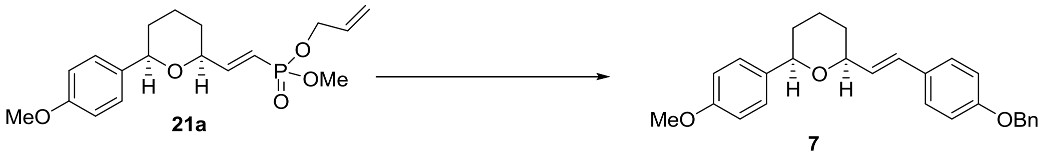

(2*S*,6*R*)-2-((*E*)-4-(benzyloxy)styryl)-6-(4-methoxyphenyl)tetrahydro-2*H*-pyran (7). To a solution of allyl vinylphosphonate **21a** (32 mg, 0.091 mmol) and 4-benzyloxystyrene (38 mg, 0.182 mmol) in dry CH_2Cl_2 (1.5 mL) was added Grubbs 2nd generation catalyst (7 mg, 0.009 mmol) followed by CuI (2 mg, 0.009 mmol) to give, after chromatography, pure **7** (18 mg, 50%).

^{1}H NMR ($CDCl_3$) δ 7.27 (11H, m), 6.93 (5H, m), 6.58 (1H, d, J_{HH} = 16.0 Hz), 6.18 (1H, dd, J_{HH} = 16.0, 5.9 Hz), 5.07 (2H, s), 4.44 (1H, br d, J_{HH} = 9.9 Hz), 4.18 (1H, m), 3.81 (3H, s), 2.01 (1H, m), 1.78 (3H, m), 1.55 (3H, m); ^{13}C NMR ($CDCl_3$) δ 159.0, 158.5, 137.2, 135.8, 130.3, 129.4, 129.2, 128.8, 128.2, 127.8, 127.7, 127.5, 115.0, 113.9, 79.8, 79.1, 70.2, 55.5, 33.6, 31.9, 24.3.

Spectral data for 27

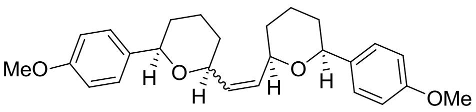

23

1-((2*R*,6*S*)-6-(4-methoxyphenyl)tetrahydro-2*H*-pyran-2-yl)-2-((2*S*,6*R*)-6-(4-methoxyphenyl)tetrahydro-2*H*-pyran-2-yl)ethene (**27**)**.** IR (neat, NaCl) 2931, 1723, 1611 cm^{-1}; ^{1}HNMR ($CDCl_3$) δ 7.31 (2H, d, J_{HH} = 8.8 Hz), 6.88 (2H, d J_{HH} = 8.8 Hz), 5.82 (1H, m), 4.34 (1H, br d, J_{HH} = 11.9, 2.0 Hz), 4.04 (1H, m), 3.81 (3H, s), 2.00 (1H,m), 1.77 (3H, m), 1.54 (1H, m), 1.40 (1H, m); ^{13}CNMR ($CDCl_3$) δ 159.1, 135.7, 127.4, 113.9, 79.7, 78.3, 55.5, 33.6, 31.6, 24.2; HRMS (FAB, NBA/NaI, MNa^+) calcd for $C_{26}H_{32}O_4Na$: 431.2198, found 431.2191.

Experimental procedure for 16b from 14b

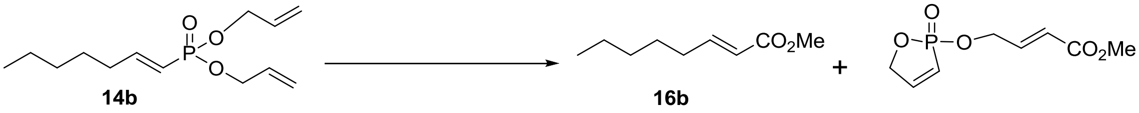

(*E*)-Methyl oct-2-enoate (16b) from reaction of diallyl phosphonate (14b). Diallyl vinylphosphonate **14b** (29.4 mg, 0.11 mmol), methyl acrylate (19 uL, 0.23 mmol, 2 eq), Grubbs 2nd generation catalyst (9 mg, 0.011 mmol, 0.1 eq), and copper(I) iodide (2 mg, 0.011 mmol, 0.1

eq) were dissolved in CH_2Cl_2 (1.1 mL). The reaction flask was fitted with a reflux condenser and placed in an oil bath pre heated to 50 °C. After 16 h, the reaction was cooled and filtered through silica gel (2 mL). The silica was rinsed with CH_2Cl_2 until just before the first dark band began eluting. The solvent was gently evaporated under reduced pressure without heating to give the crude product (128 mg). The crude product was purified by column chromatography (SiO_2, 15% CH_2Cl_2 in pentane) to give a solution of the ester **16b** in CH_2Cl_2 (39.4 mg). The yield of ester (7.7 mg, 45% yield) was estimated from the ^{1}H NMR spectrum.

Experimental procedure and spectral data for 24 and 32

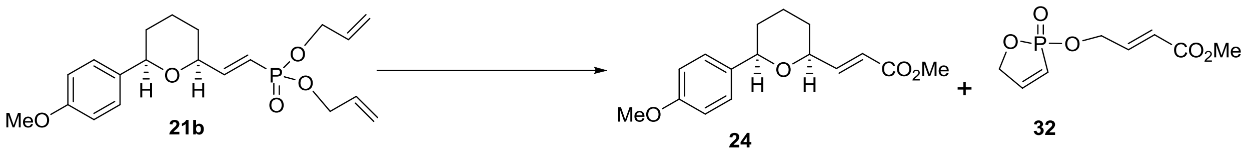

(*E*)-methyl 3-((2*S*,6*R*)-6-(4-methoxyphenyl)tetrahydro-2*H*-pyran-2-yl)acrylate (**20**) **from reaction of diallyl phosphonate (21b)**. To a solution of di-allyl vinylphosphonate **21b** (0.021 g, 0.056 mmol) and methyl acrylate (0.01 mL, 0.11 mmol) in dry CH_2Cl_2 (0.9 mL) was added Grubbs 2nd generation catalyst (0.005 g, 0.0056 mmol) followed by CuI (0.001 g, 0.0056 mmol) to give, after chromatography, pure ester **20** (0.009 g, 58%). Further elution gave **(*E*)-methyl 4-((2-oxido-1,2-oxaphosphol-2(5*H*)-yl)oxy)but-2-enoate (32).** ^{1}HNMR ($CDCl_3$) δ 7.20 (1H, ddt, J_{HH} = 8.6, 1.6 Hz, J_{HP} = 46.9 Hz), 6.96 (1H, ddt, J_{HH} = 15.6, 4.1 Hz, J_{HP} = 0.9 Hz), 6.22 (1H, ddt, J_{HH} = 8.5, 2.4 Hz J_{HP} = 34.5 Hz), 6.11 (1H, ddt, J_{HH} = 15.7, 2.0 Hz, J_{HP} = 4.0 Hz), 4.80 (4H, m), 3.76 (3H, s); ^{13}C NMR ($CDCl_3$) δ 166.4, 149.2 (d, J_{CP} = 17.6 Hz), 142.2 (J_{CP} = 6.2 Hz), 121.8, 116.7 (d, J_{CP} = 163.6 Hz), 71.0 (d, J_{CP} = 13.4 Hz), 65.1 (d , J_{CP} = 5.8 Hz), 52.0; ^{31}P NMR ($CDCl_3$) δ 42.8.

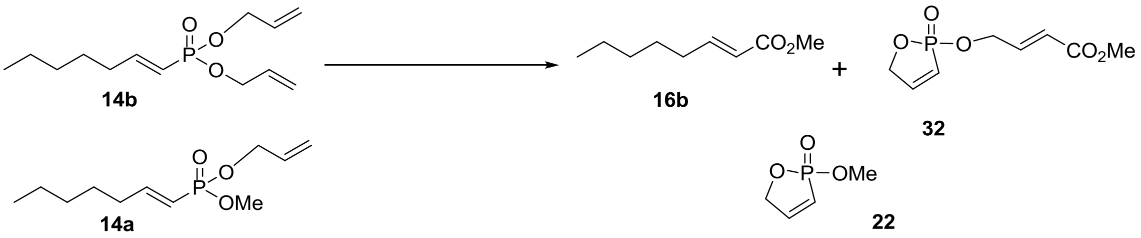

(*E*)-Methyl oct-2-enoate (16b) from reaction of mono and diallyl phosphonate (14 and 14b). A mixture of mono allyl phoshonate **14a** (60 mg, 0.25 mmol) and diallyl phosphonate **14b** (26 mg, 0.10 mmol), methyl acrylate (63 uL, 0.7 mmol, 2 equiv), Grubbs 2nd generation catalyst (30 mg, 0.035 mmol, 0.1 equiv.) and copper(I) iodide (7 mg 0.035 mmol, 0.1 equiv) were dissolved in CH_2Cl_2 (3.5 mL). The reaction flask was fitted with a reflux condenser and placed in an oil bath pre heated to 50 °C. After 16 h, the reaction was cooled and filtered through silica gel (2 mL). The silica was rinsed with CH_2Cl_2 until just before the first dark band began eluting. The solvent was gently evaporated under reduced pressure without heating to give the crude product (177 mg). The crude product was purified by column chromatography (SiO_2, 15% CH_2Cl_2 in pentane) to give a solution of the ester **16b** in CH_2Cl_2 (76.7 mg). The yield of ester (46.9 mg, 86% yield) was estimated from the ^{1}H NMR spectrum.

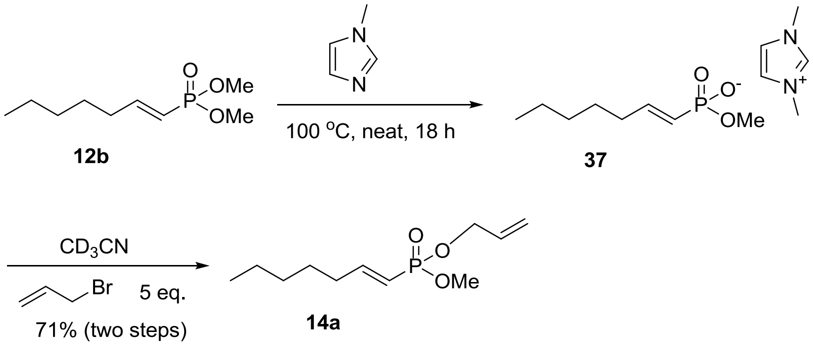

Selective mono allylation to form 14a. Vinylphosphonate **12b** (102.2 mg 0.496 mmol, 1 eq.) and *N*-methylimidazole (49.4 uL, 50.9 mg, 0.602 mmol, 1.25 eq.) were added to an NMR tube which was heated to 100 ^{o}C for 18 h. The resulting oil was transferred to a vial using CD_3CN as solvent. The solvent was evaporated and the oil was washed with Et_2O (2 x 4 mL) to remove any remaining starting materials and then dried in vacuo to give the imidazolium salt (139 mg, 97%). The salt was transferred to a NMR tube with CD_3CN (0.5 mL) and allyl bromide (210 uL, 300 mg, 2.48 mmol, 5 eq) was added. The reaction was monitored by ^{31}P NMR and was complete after two days at room temperature. The solvent and excess allyl bromide were removed in vacuo. The oily residue was dissolved in CH_2Cl_2 (4 mL), washed with water (2 x 2 mL) and brine (2 mL), dried Na_2SO_4 and evaporated (87.2 mg, brown oil). The crude product was purified by silica gel chromatography (1:1 EtOAc/hexane, isocratic) to give the mono allyl phopshonate (77.2 mg, 71%).

References

1. Trost, B. M.; Ball, Z. T.; Jöge, T. *J. Am. Chem. Soc.* **2002,** *124*, 7922-7923.

2. Trost, B. M.; Li, C.-J. *J. Am. Chem. Soc.* **1994**, *116*, 10819-10820.

^{1}H, ^{13}C NMR and ^{31}P NMR spectra of 14a

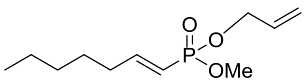

14a

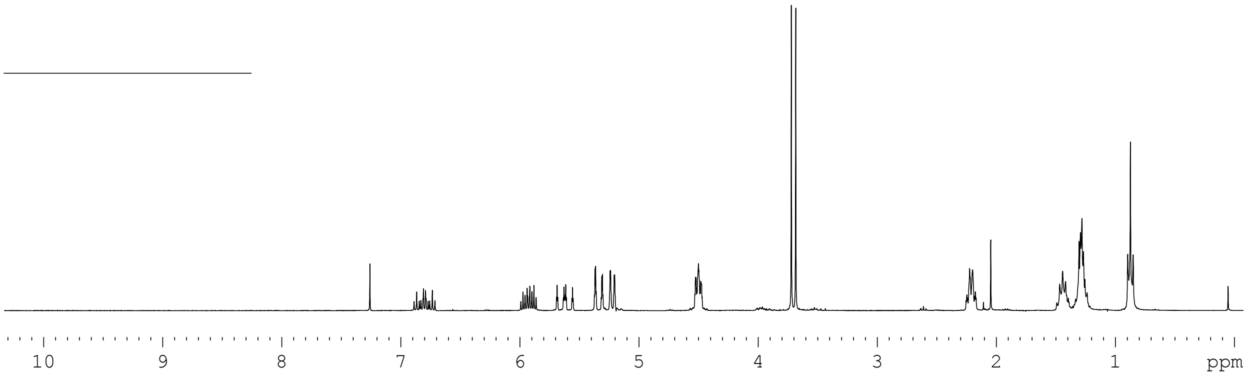

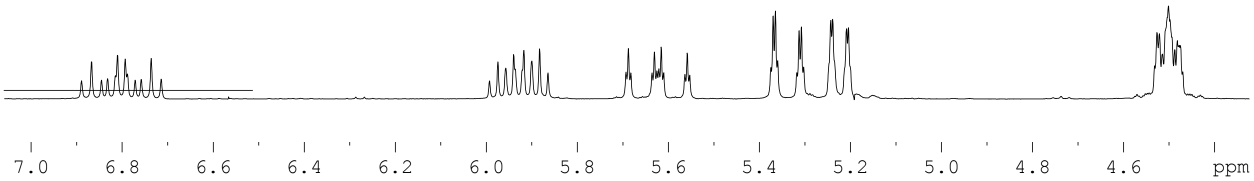

^{13}C NMR

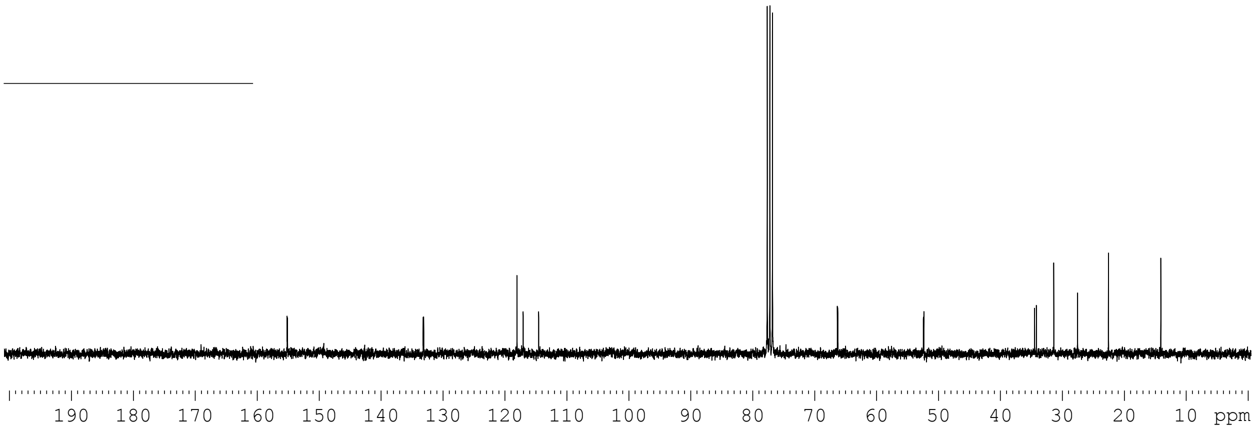

^{1}H, ^{13}C NMR and ^{31}P NMR spectra of 14a (cont)

^{13}C NMR (expansion)

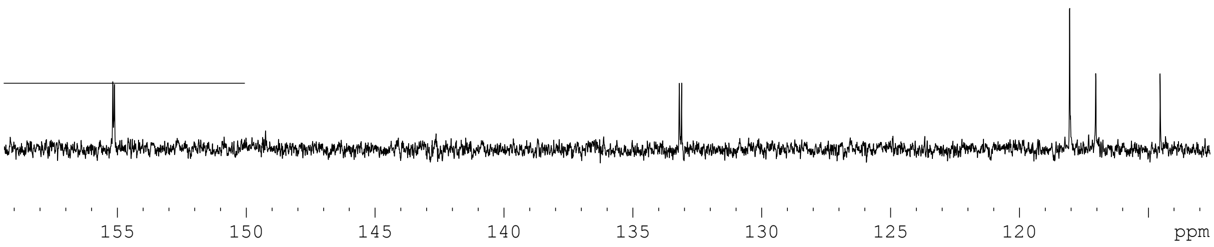

^{13}C NMR (expansion)

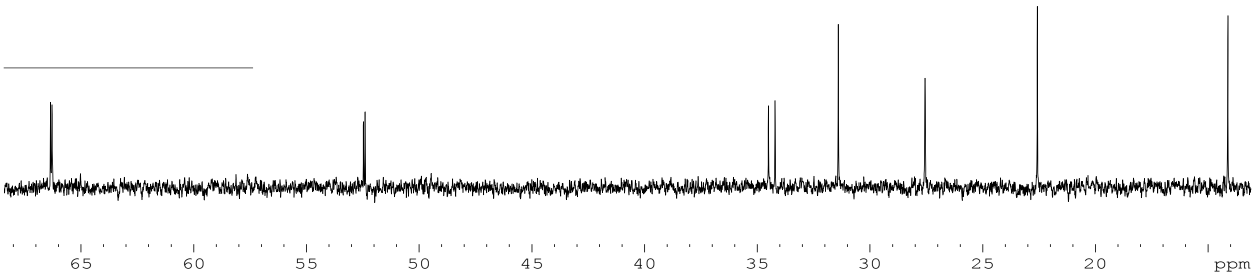

^{31}P NMR

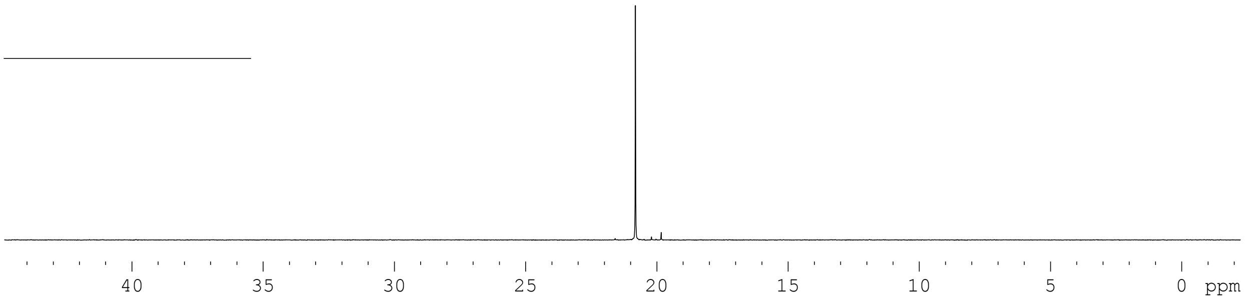

^{1}H, ^{13}C NMR and ^{31}PNMR spectra of 14b

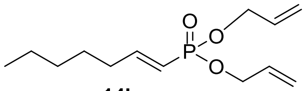

14b

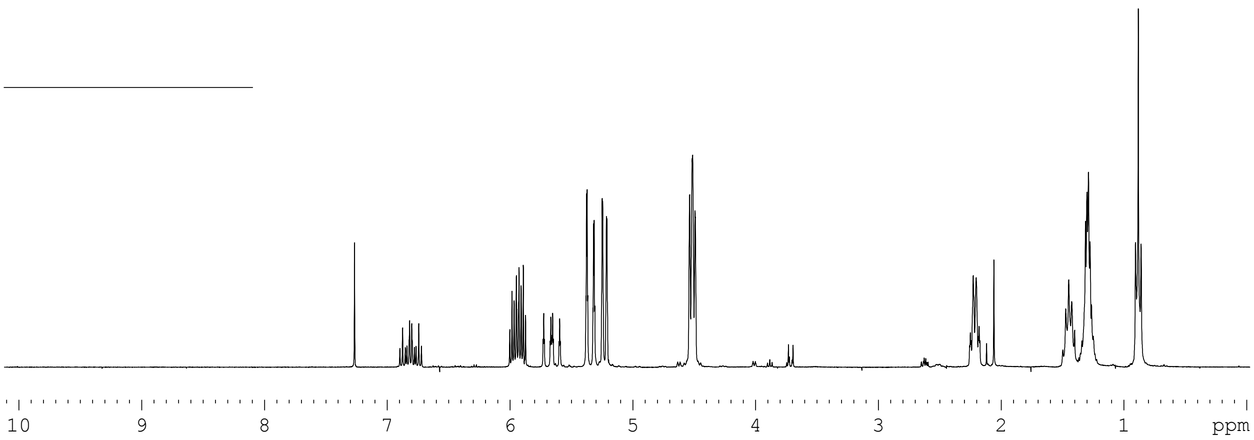

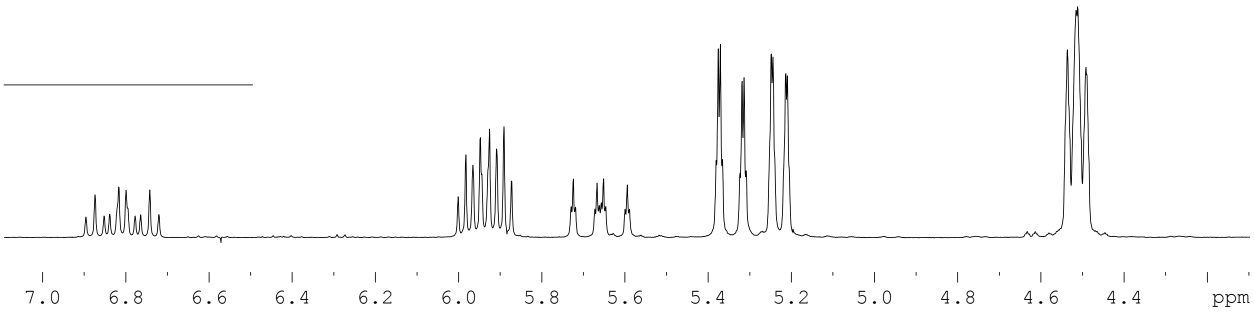

^{13}C NMR (full)

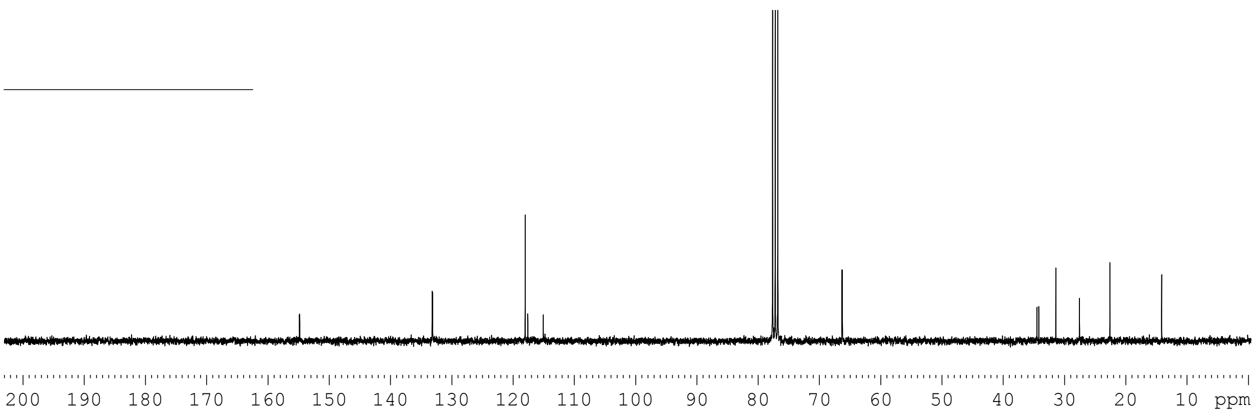

^{1}H, ^{13}C NMR and ^{31}PNMR spectra of 14b (cont)

^{13}C NMR (expansion)

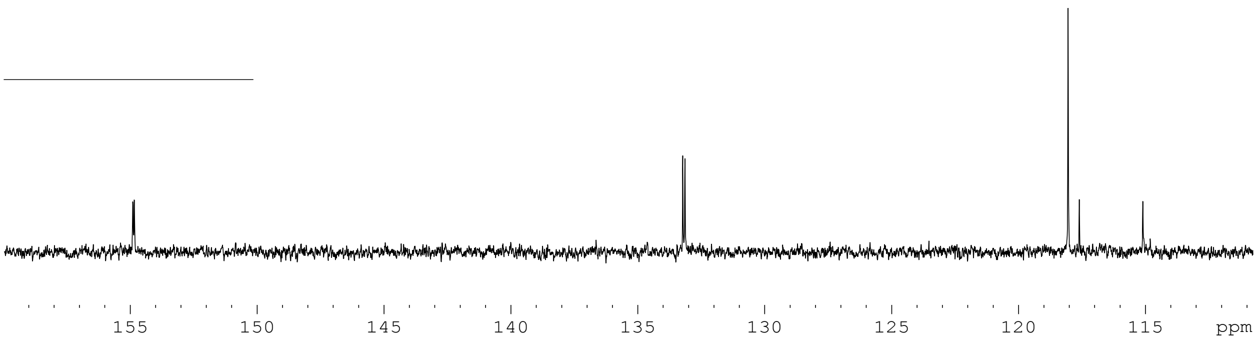

^{13}C NMR (expansion)

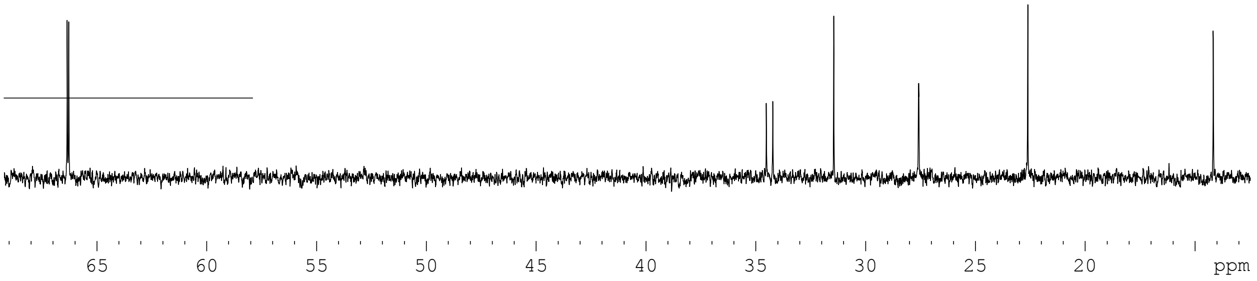

^{31}P NMR

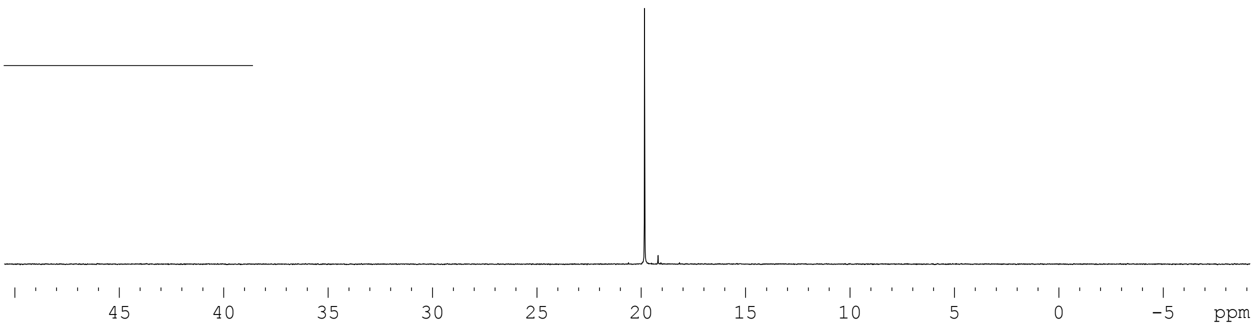

^{1}H, ^{13}C NMR and ^{31}PNMR spectra of 20a

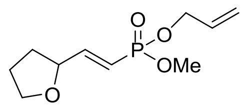

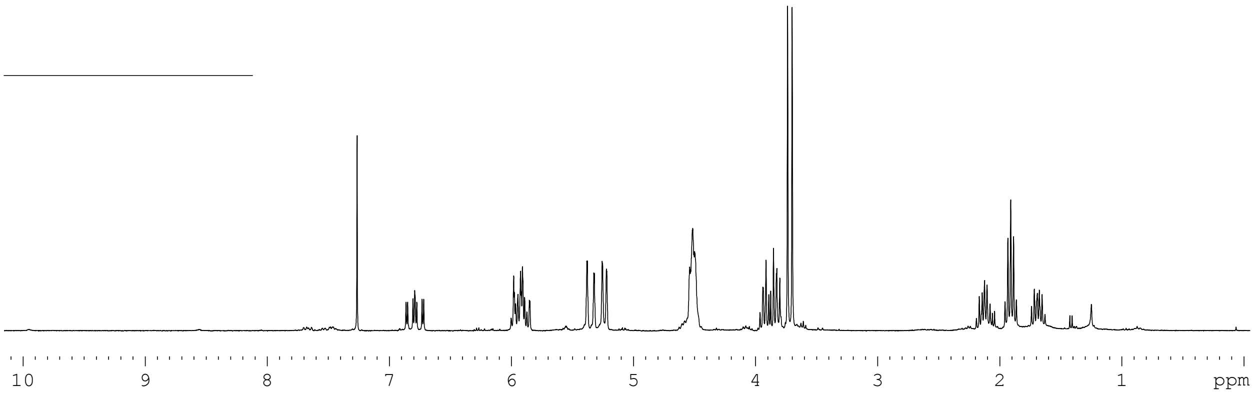

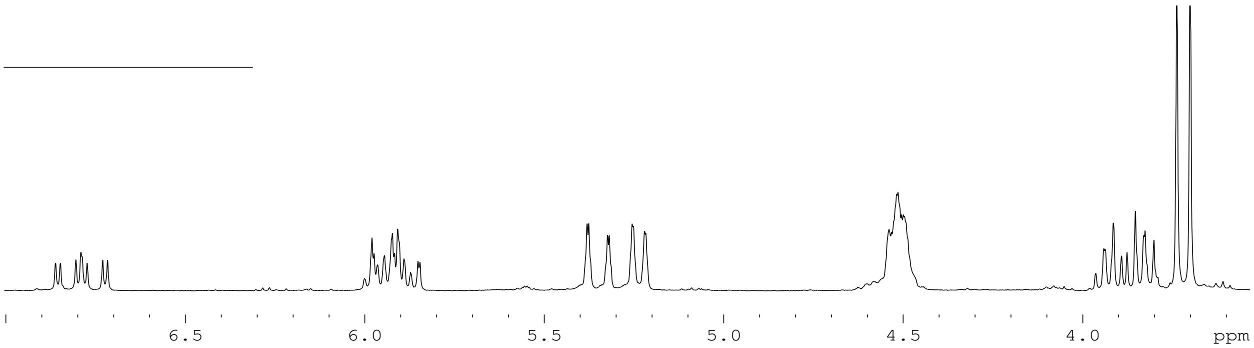

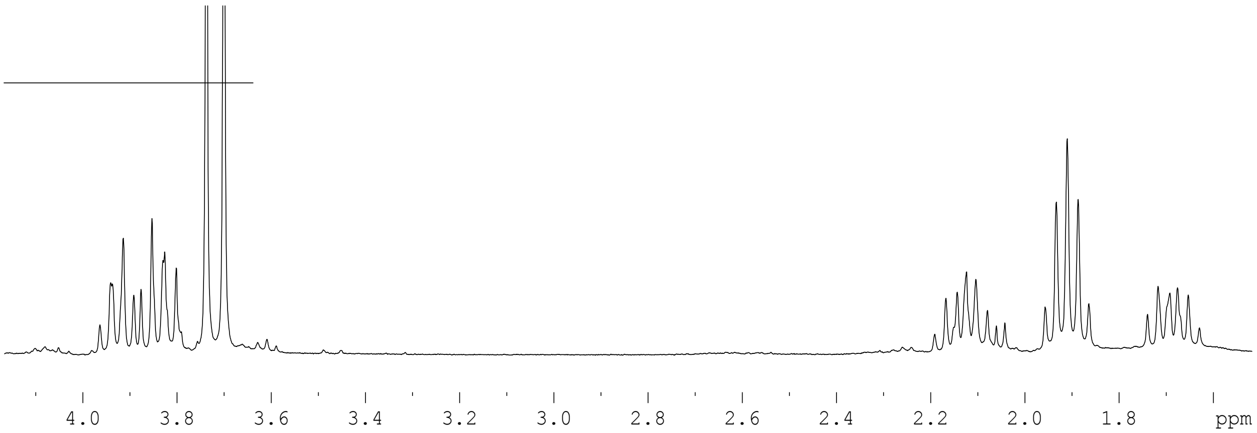

^{1}H, ^{13}C NMR and ^{31}PNMR spectra of 20a (cont)

^{13}C NMR (full)

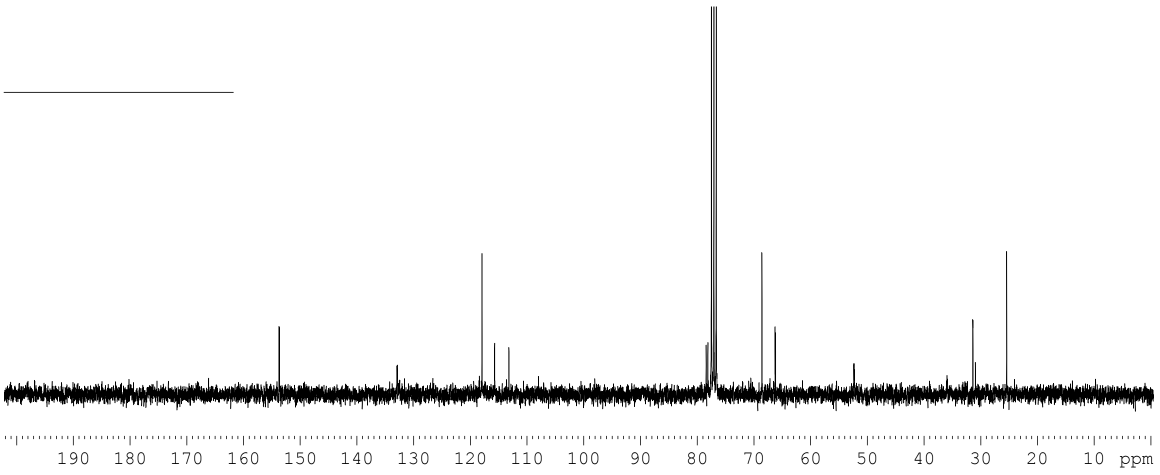

^{13}C NMR (expansion)

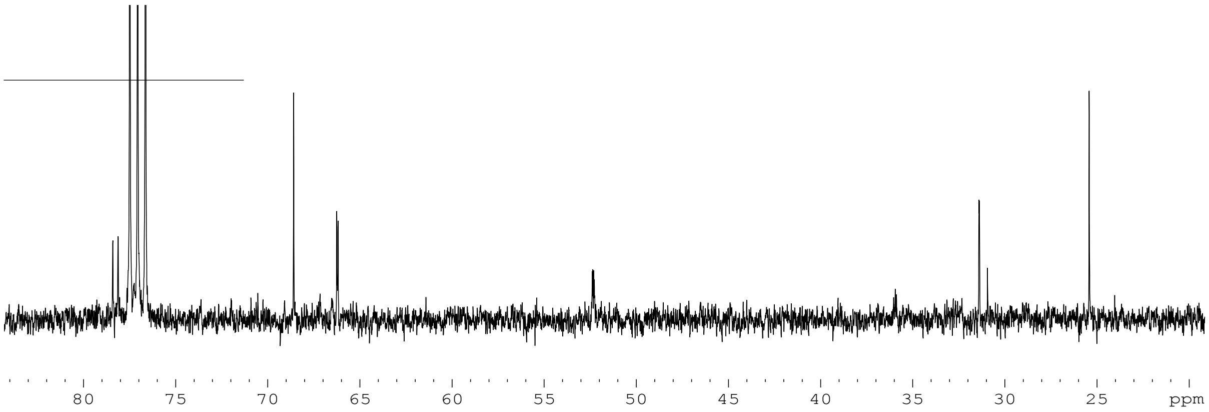

^{31}P NMR

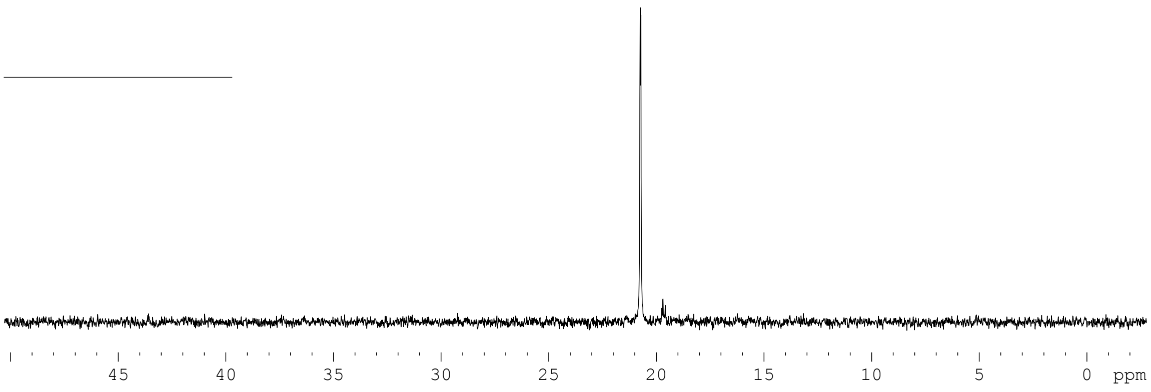

^{1}H, ^{13}C NMR and ^{31}PNMR spectra of 20b

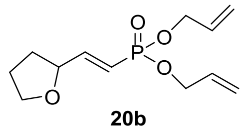

20b

with Ph_3PO

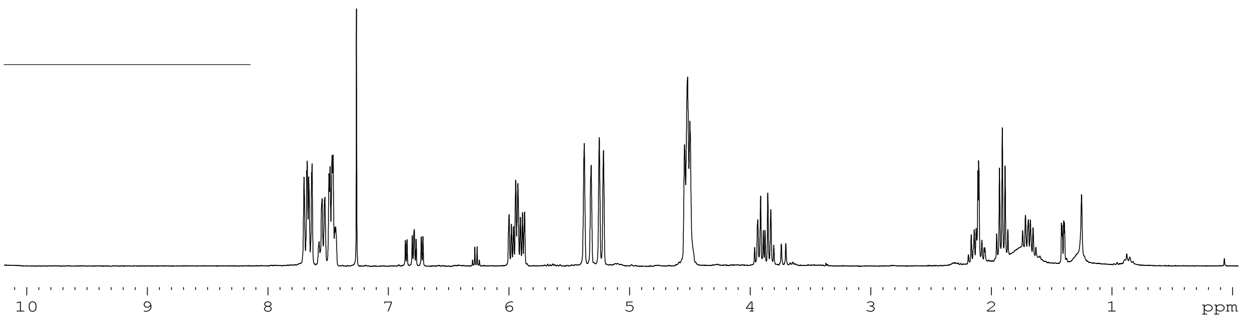

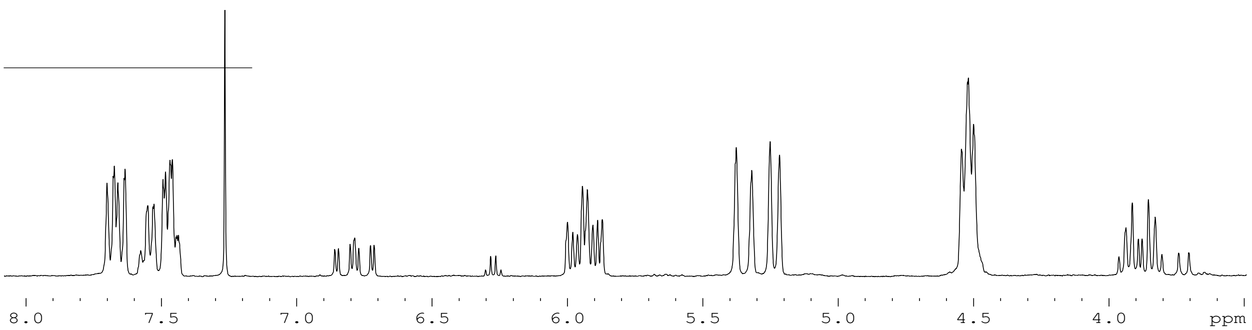

^{13}C NMR
(full)

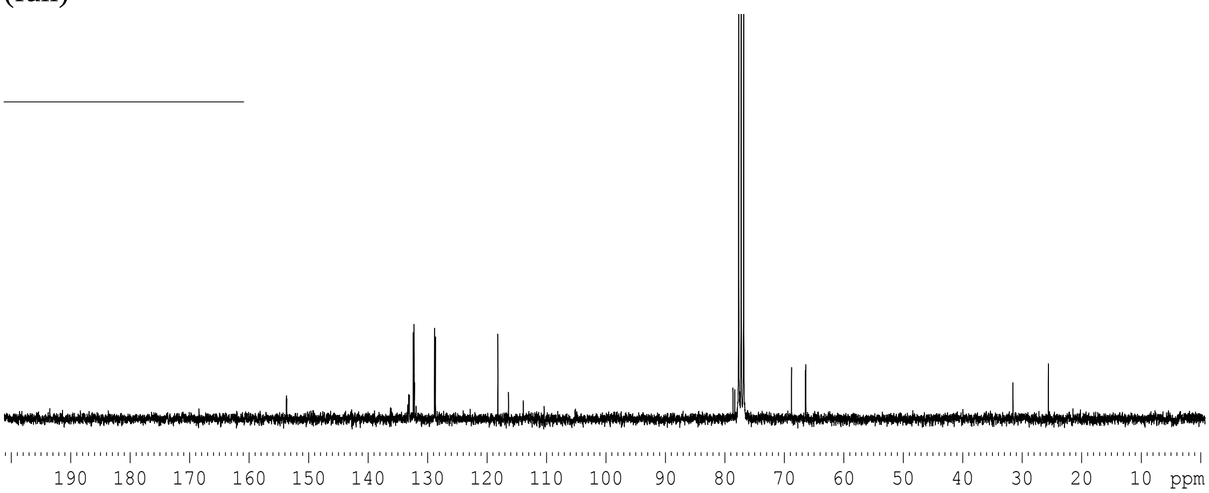

^{1}H, ^{13}C NMR and ^{31}PNMR spectra of 20b (cont)

^{13}C NMR (expansion)

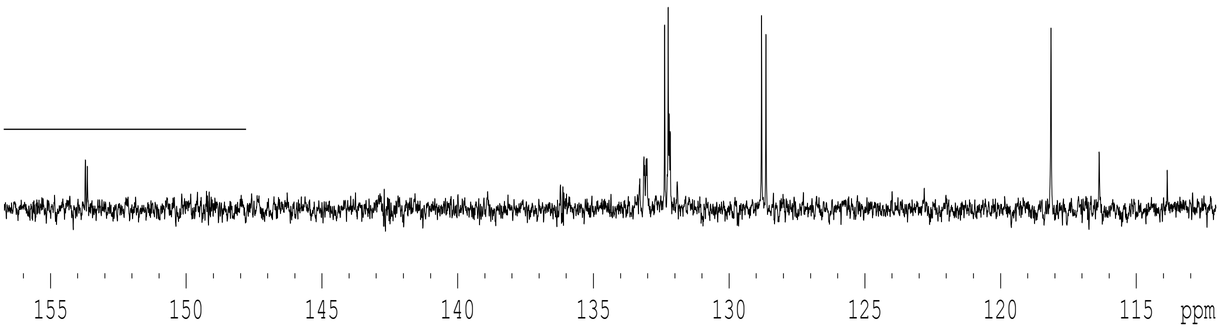

^{13}C NMR (expansion)

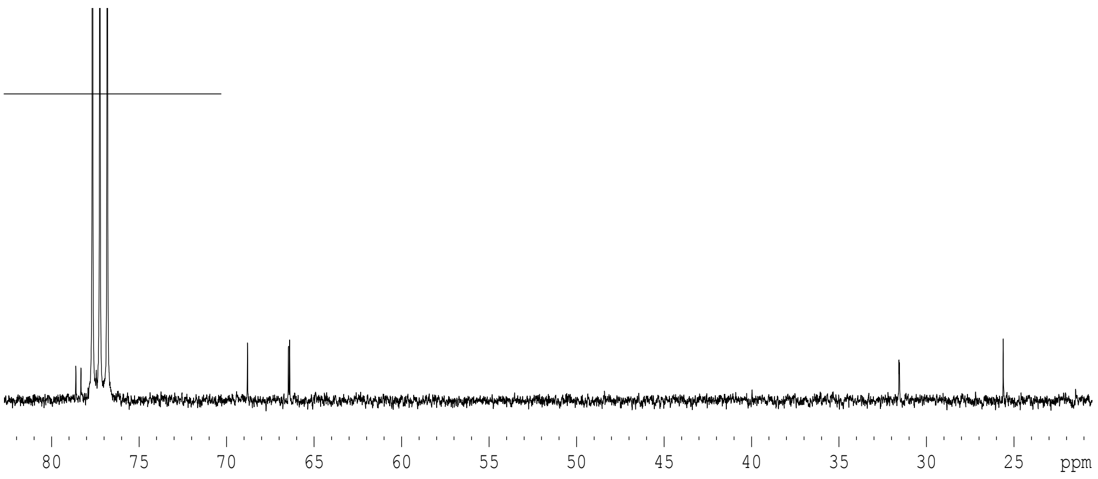

^{31}P NMR

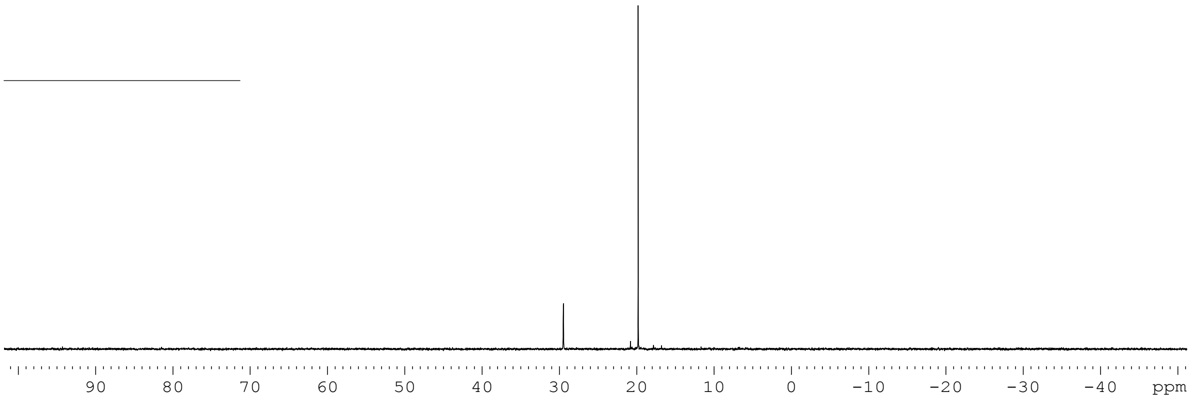

^{1}H and ^{13}C NMR spectra of 21a

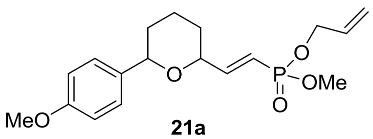

21a

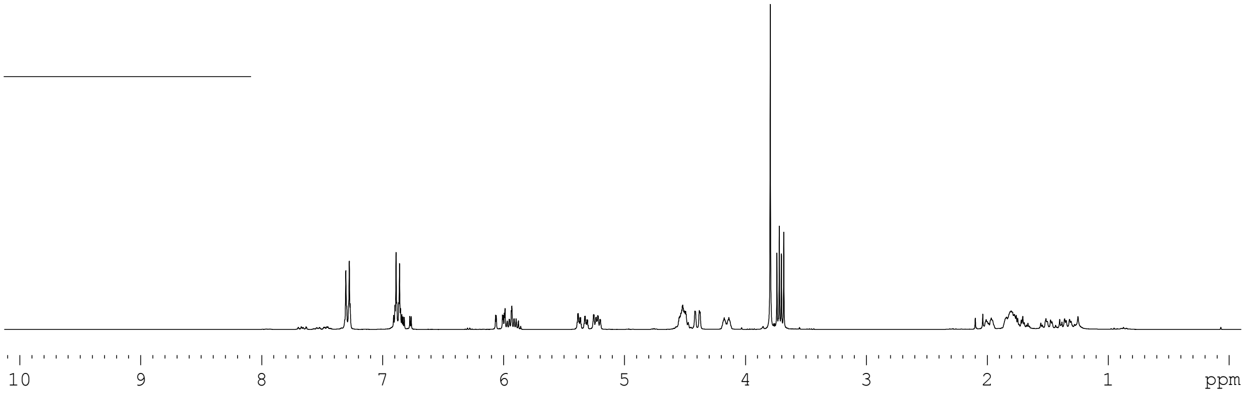

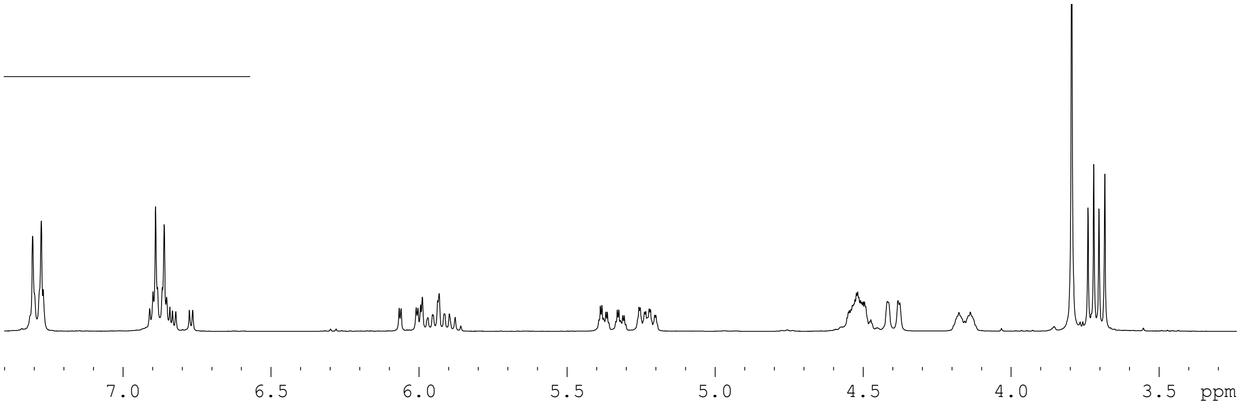

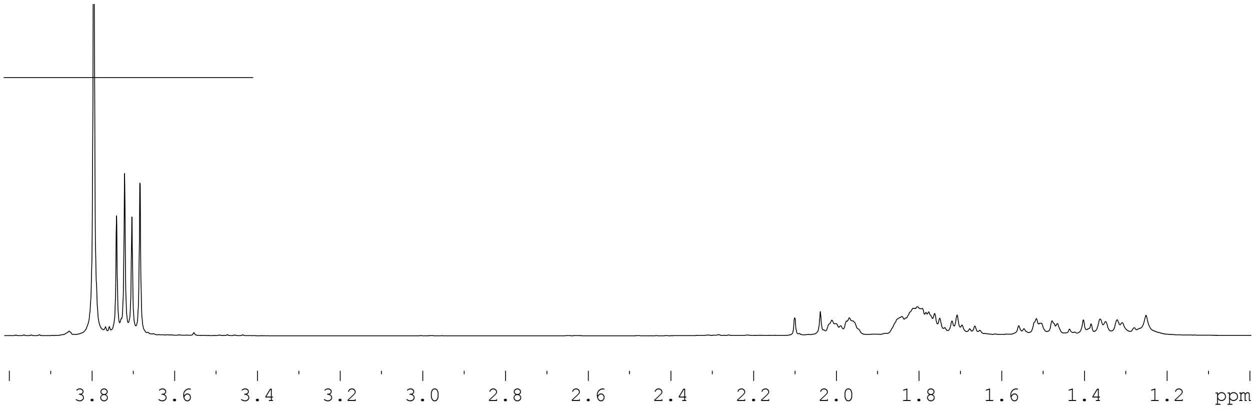

^{1}H and ^{13}C NMR spectra of 21a (cont)

^{13}C NMR (full)

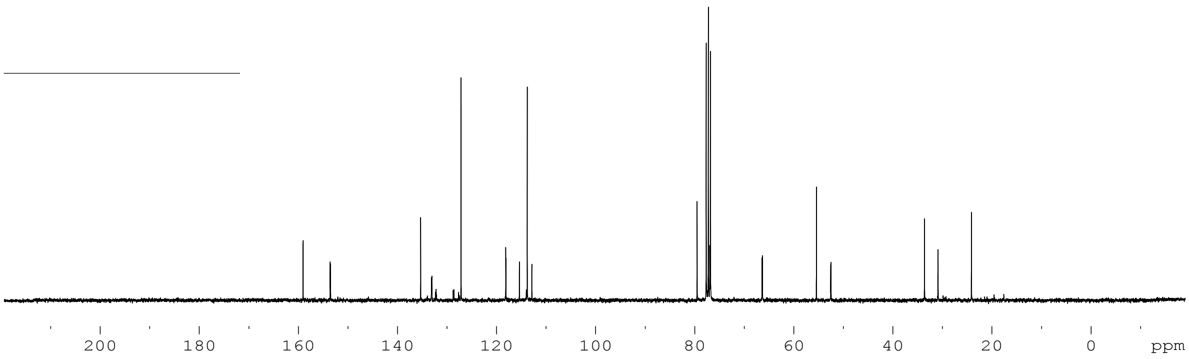

^{13}C NMR
(expansion)

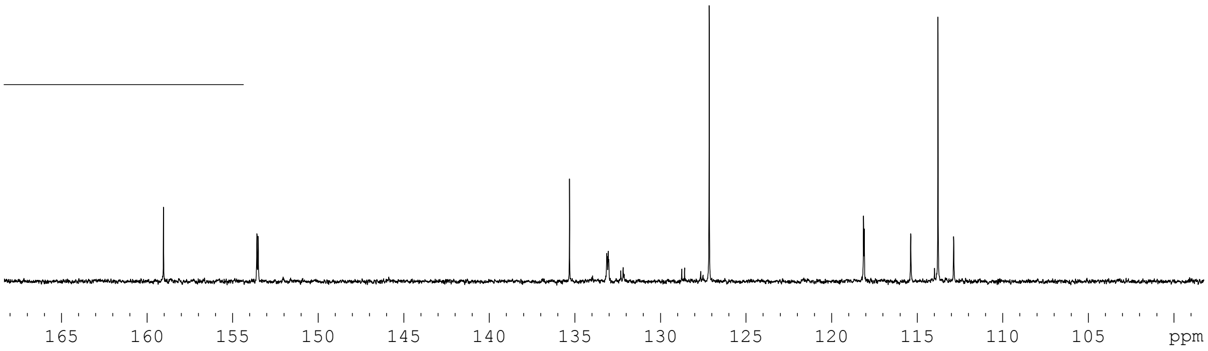

^{13}C NMR
(expansion)

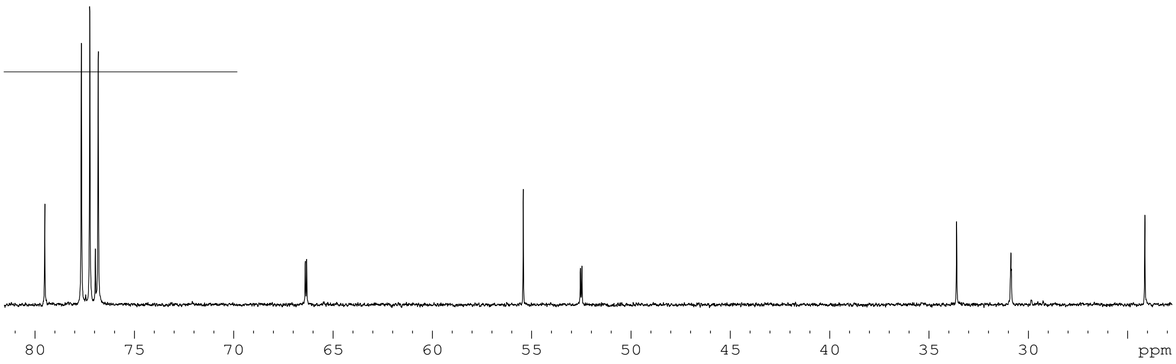

^{1}H-^{1}H COSY and ^{31}P NMR spectrum of 21a

^{31}P NMR

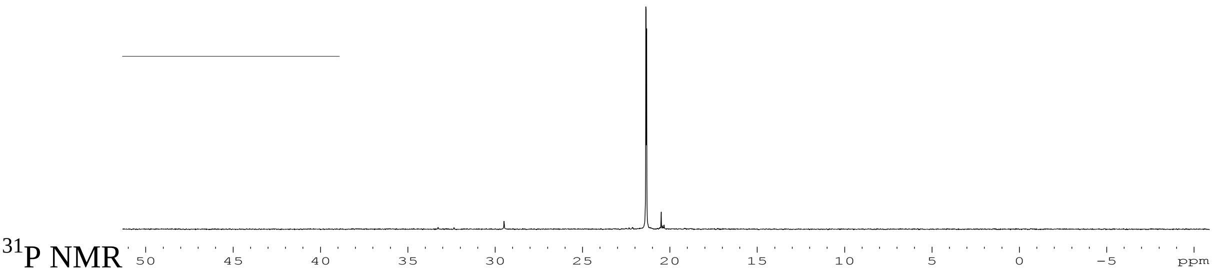

^{1}H-^{1}H COSY

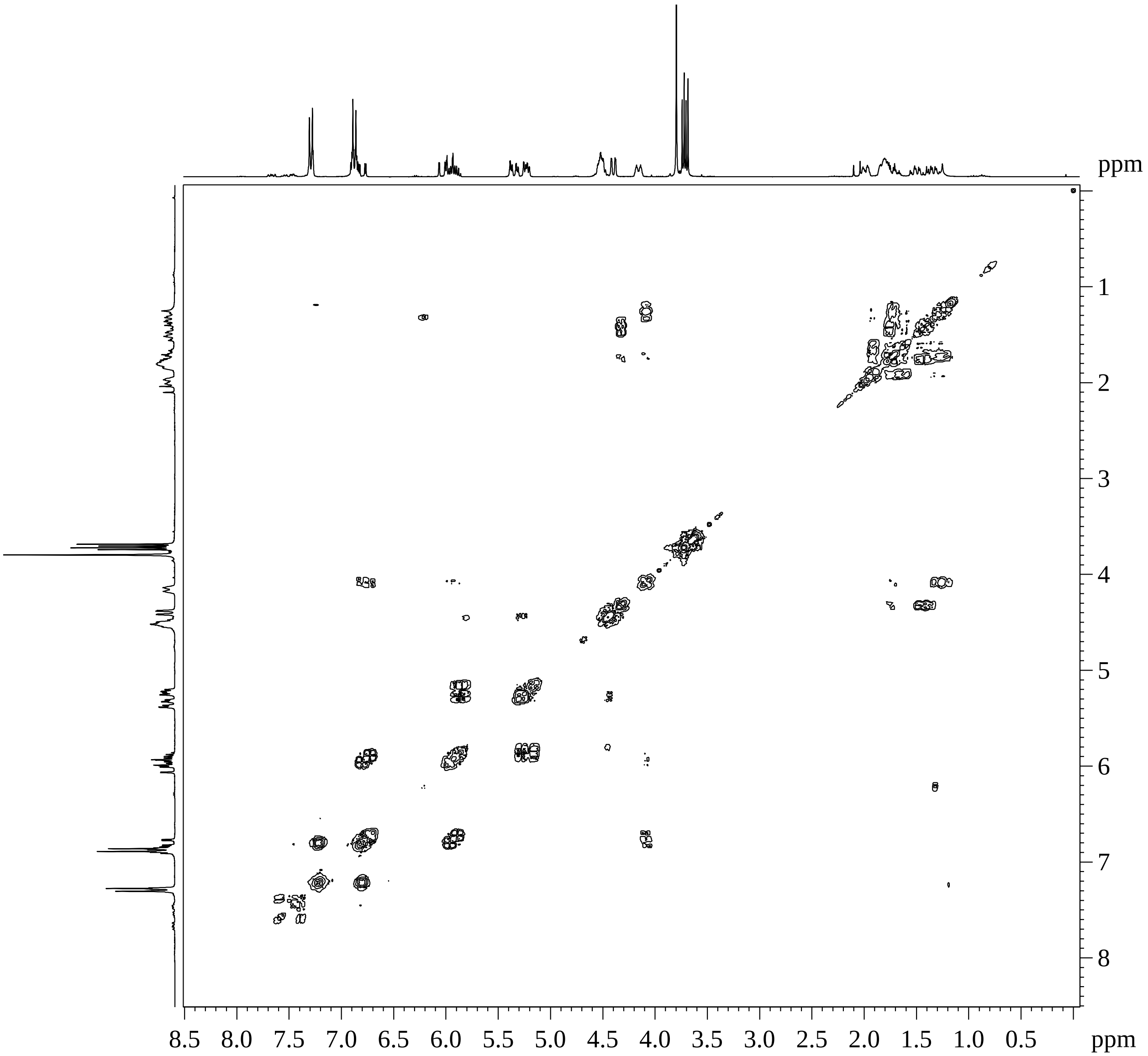

^{1}H, ^{13}C NMR and ^{31}P NMR spectra of 21b

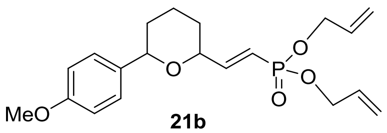

21b

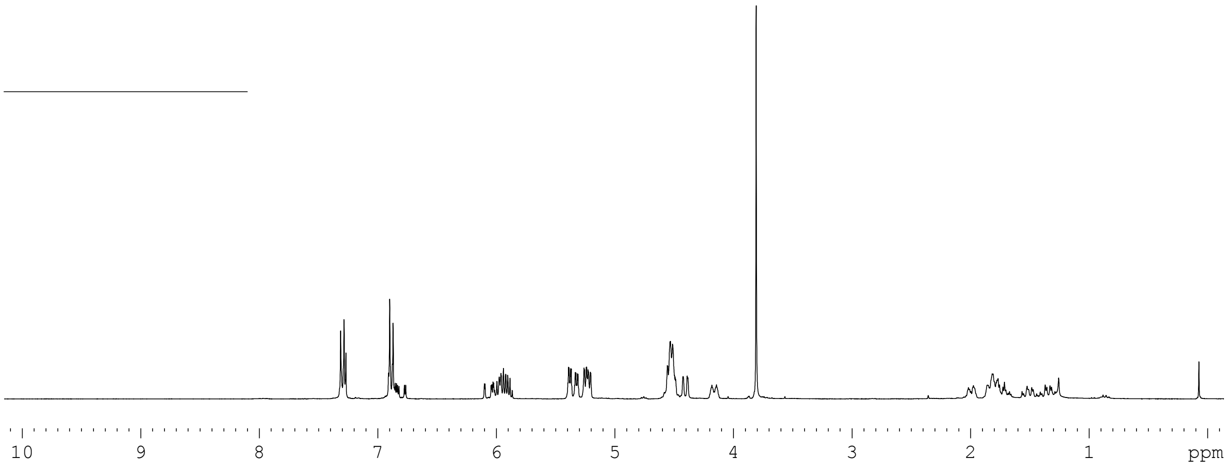

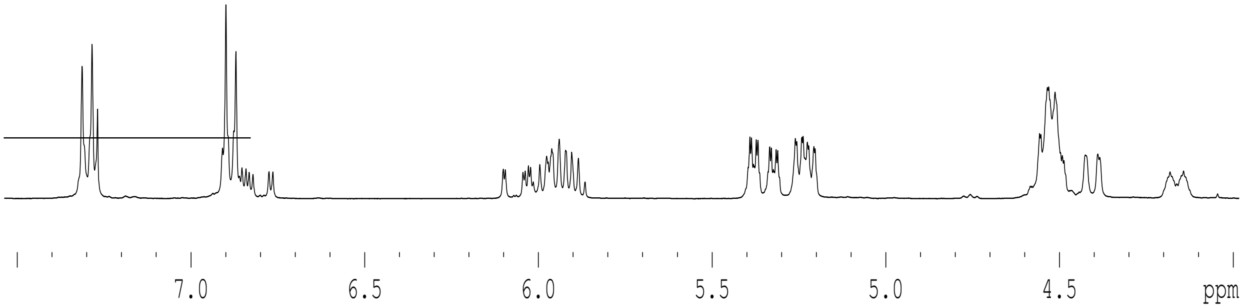

^{13}C NMR (full)

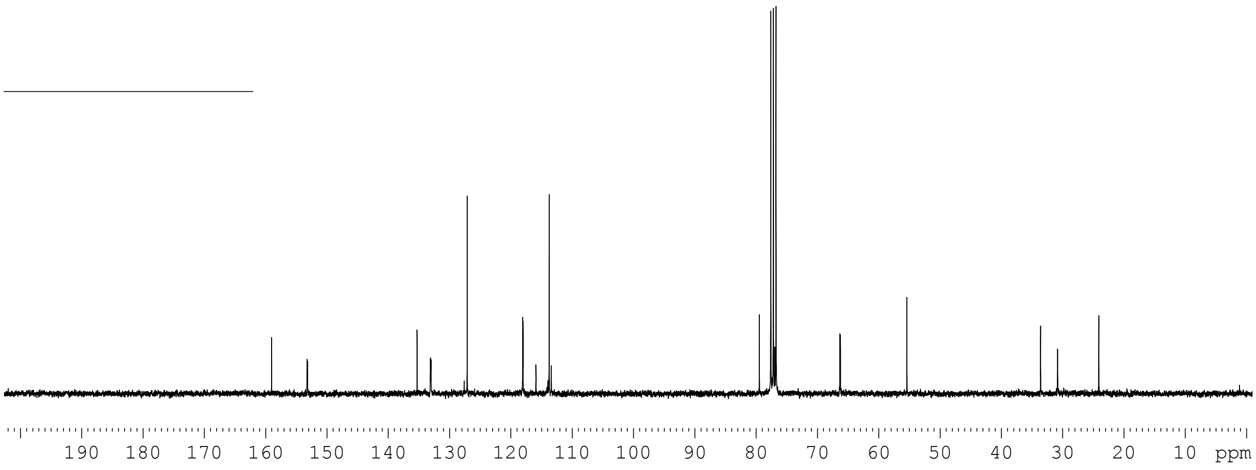

^{1}H, ^{13}C NMR and ^{31}P NMR spectra of 21b (cont)

^{13}C NMR
(expansion)

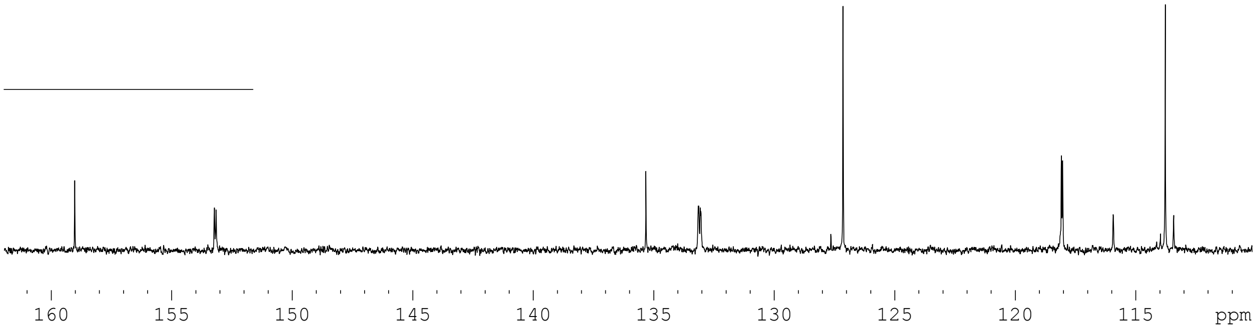

^{13}C NMR (expansion)

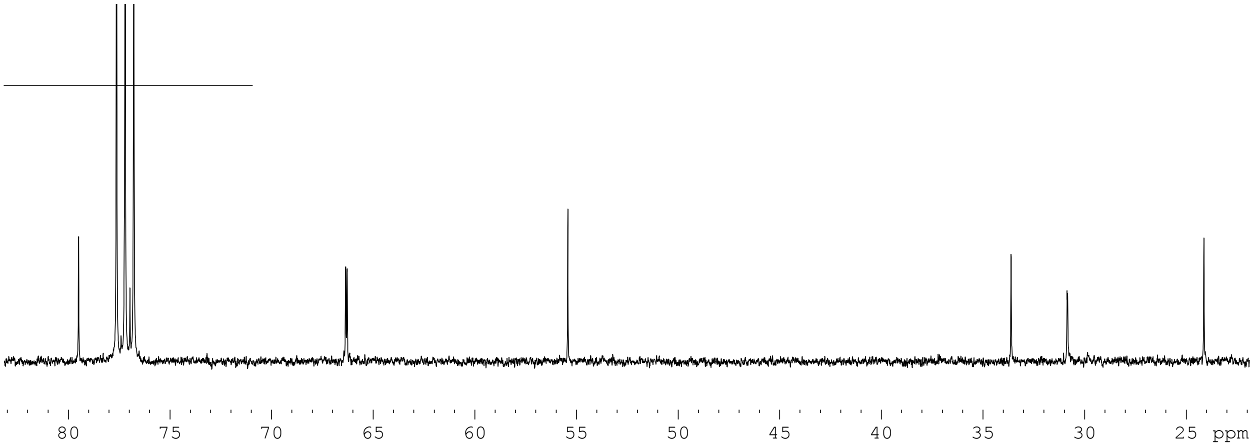

^{31}P NMR

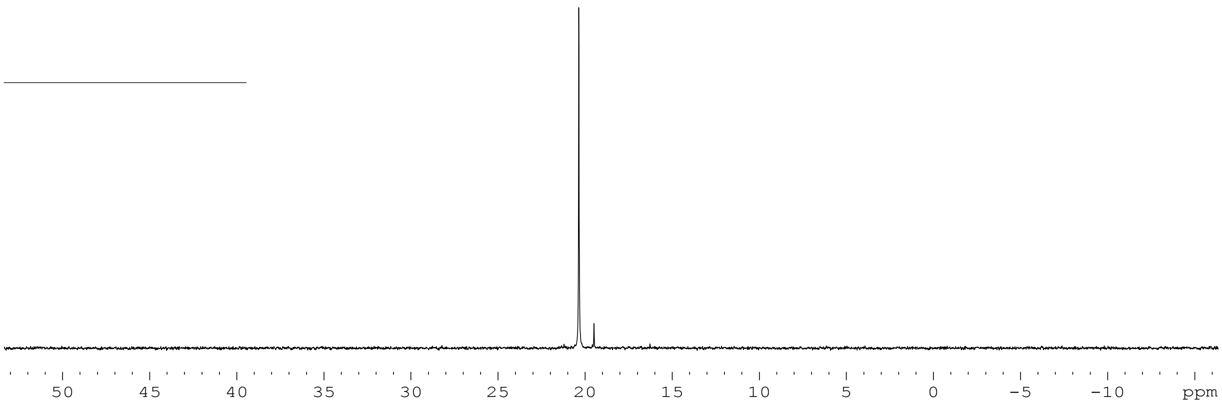

^{1}H-^{1}H COSY spectrum of 21b

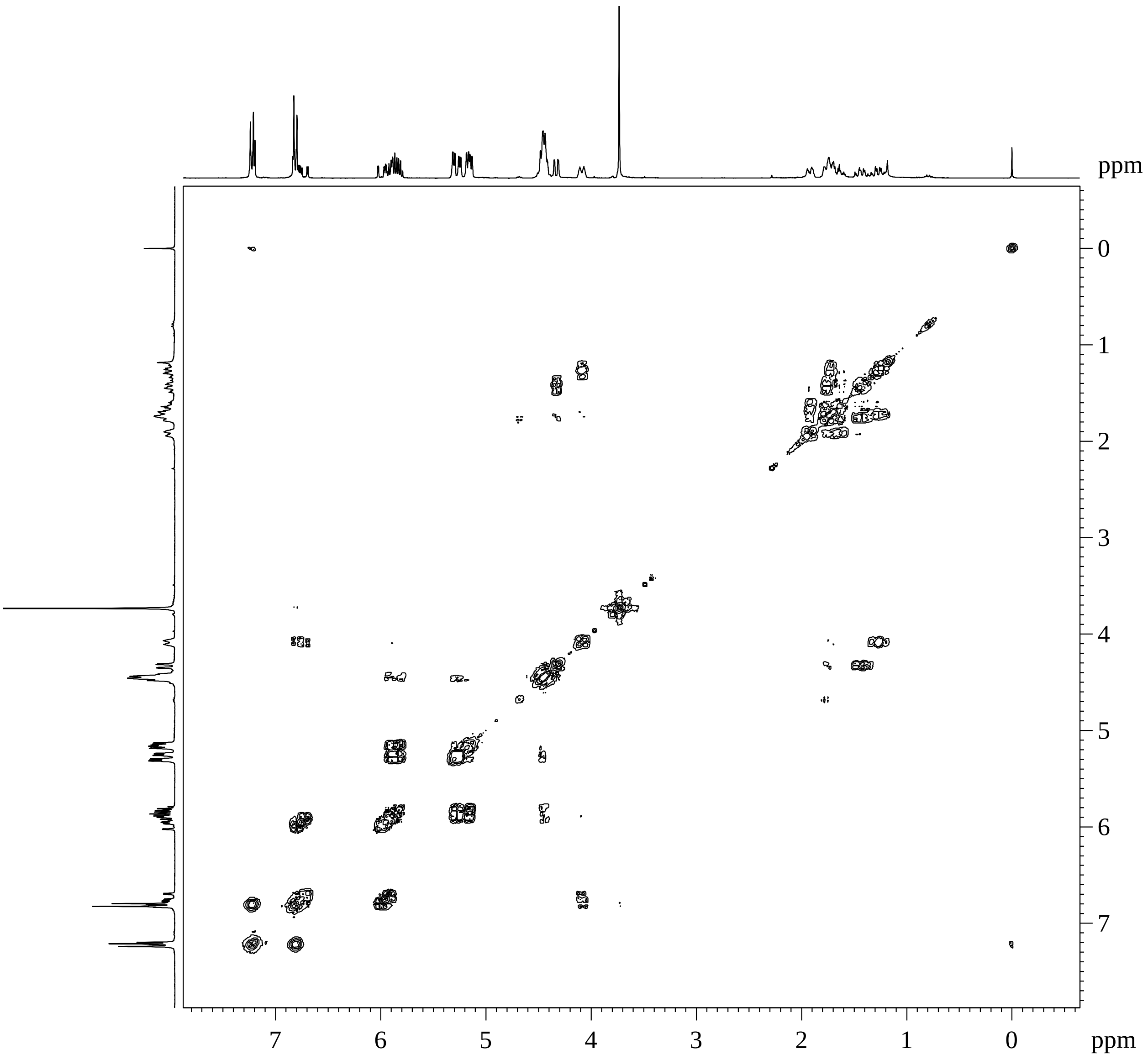

^{1}H NMR spectrum of 16b

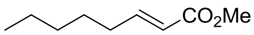

16b

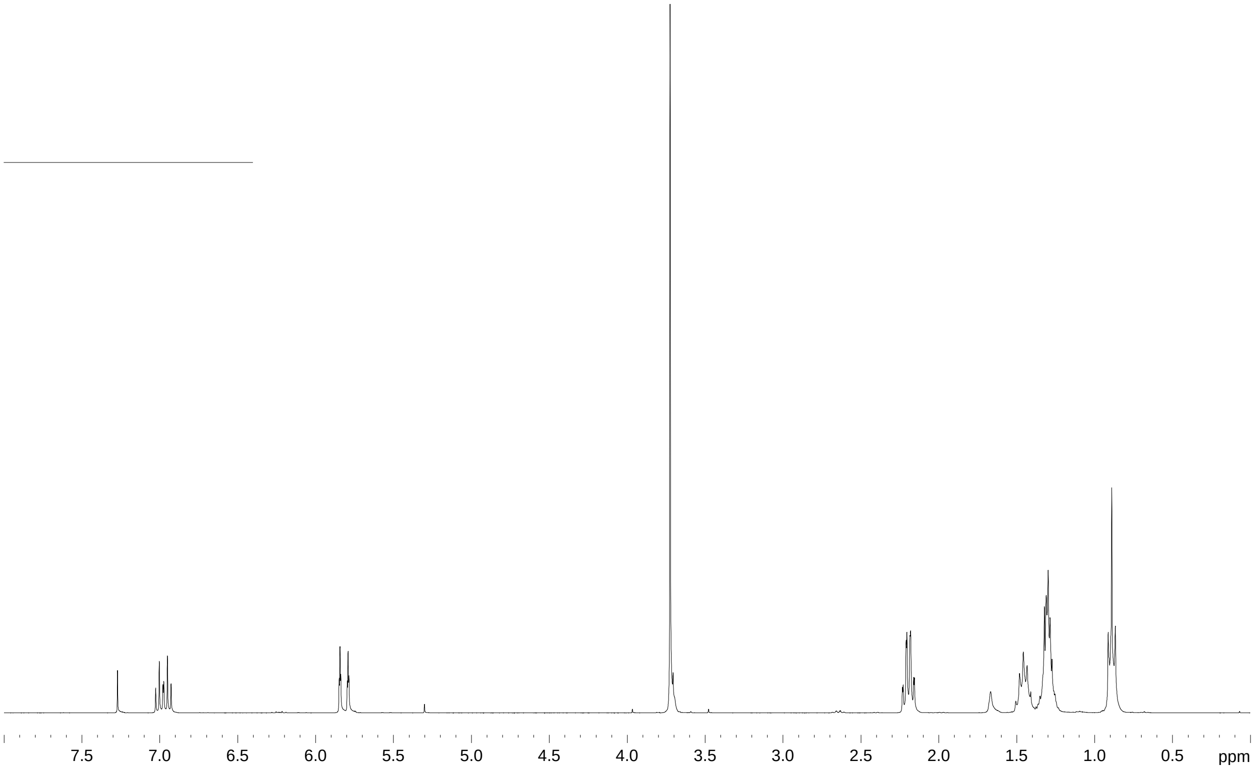

^{1}H NMR spectrum of 23

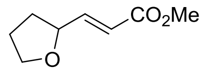

23

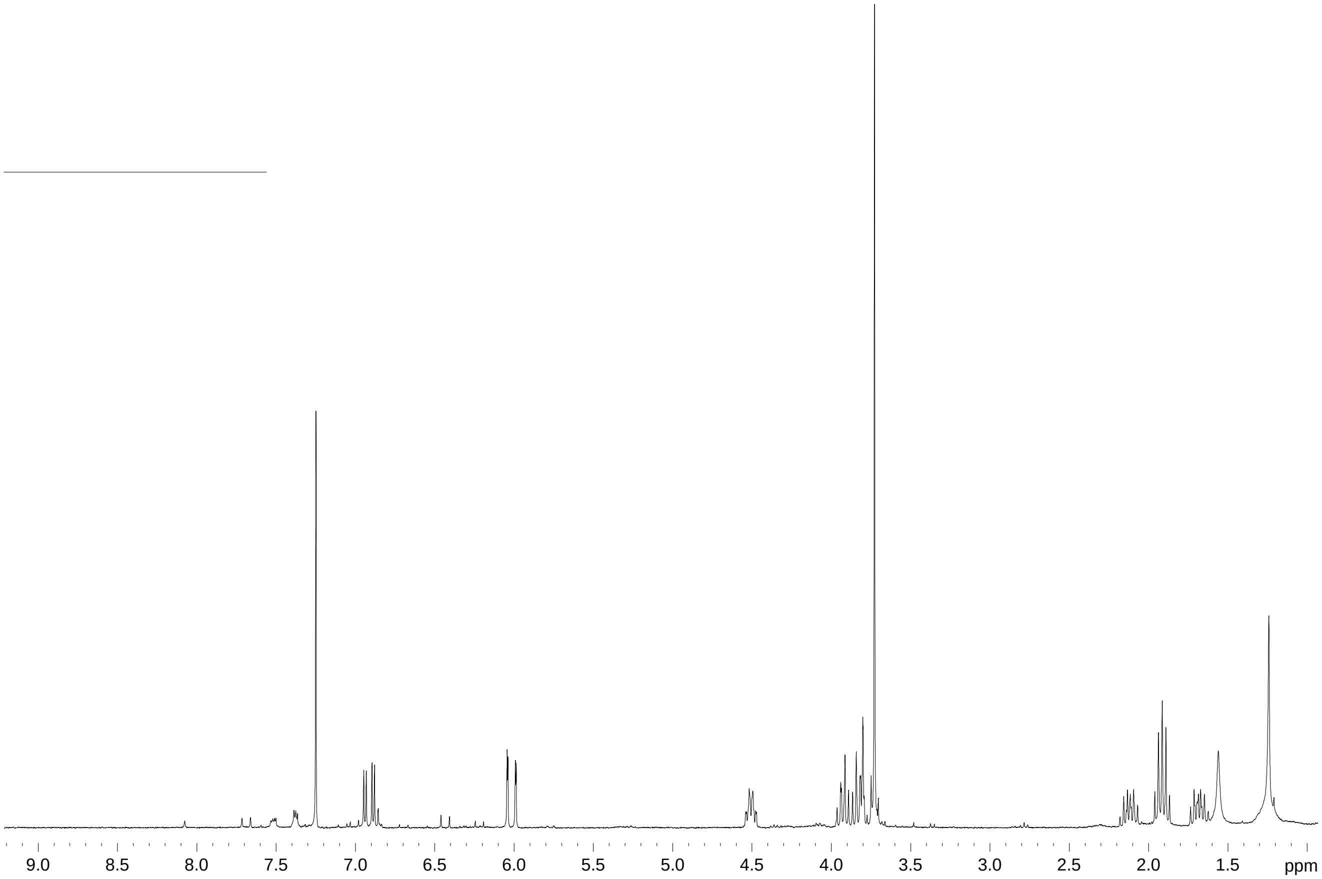

^{1}H and ^{13}C NMR spectra of 24

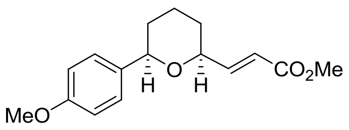

24

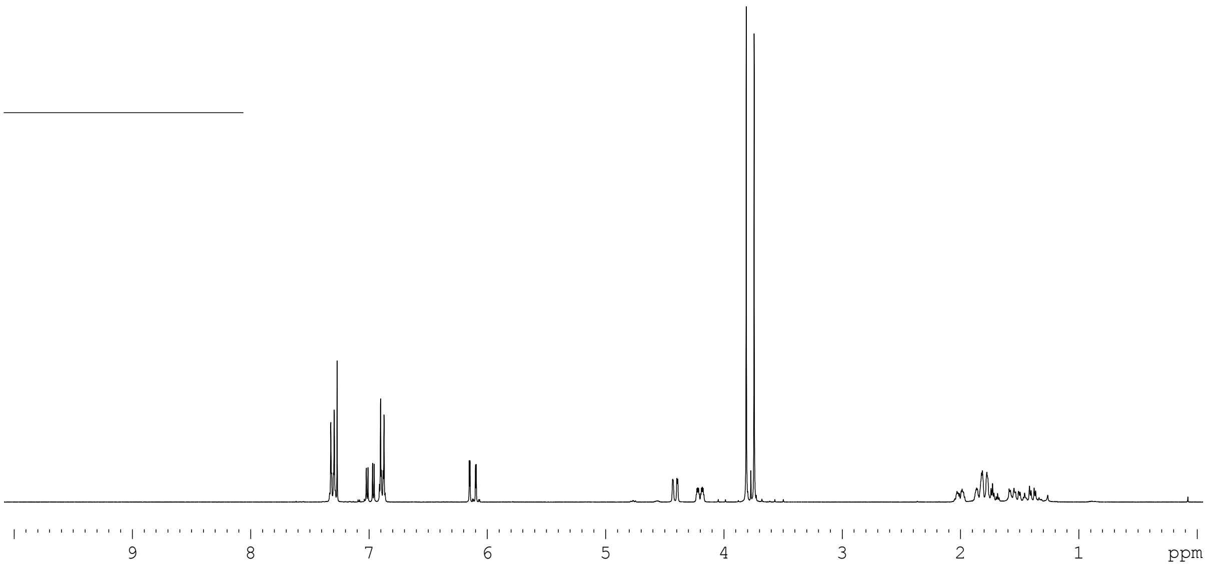

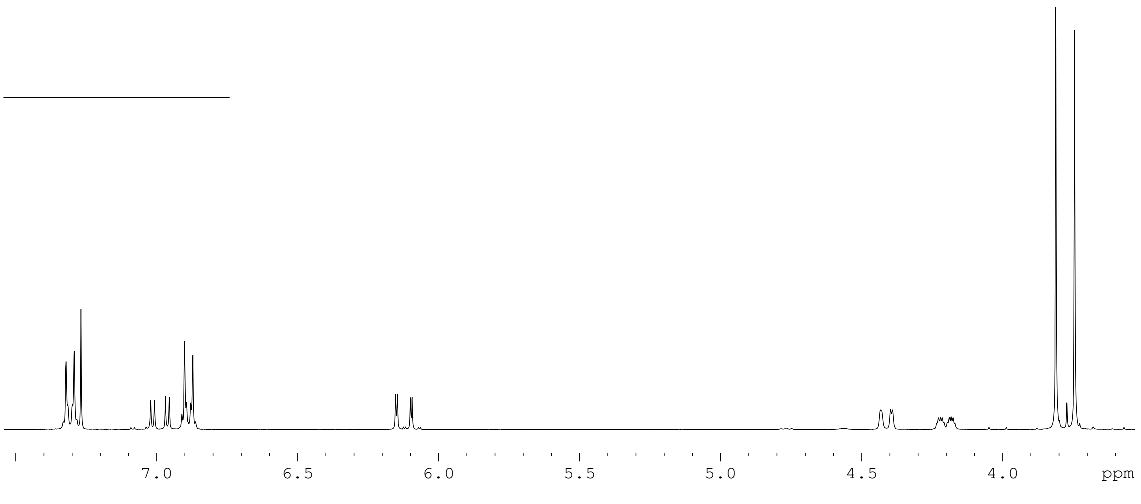

^{1}H and ^{13}C NMR spectra of 24 (cont)

^{13}C NMR (full)

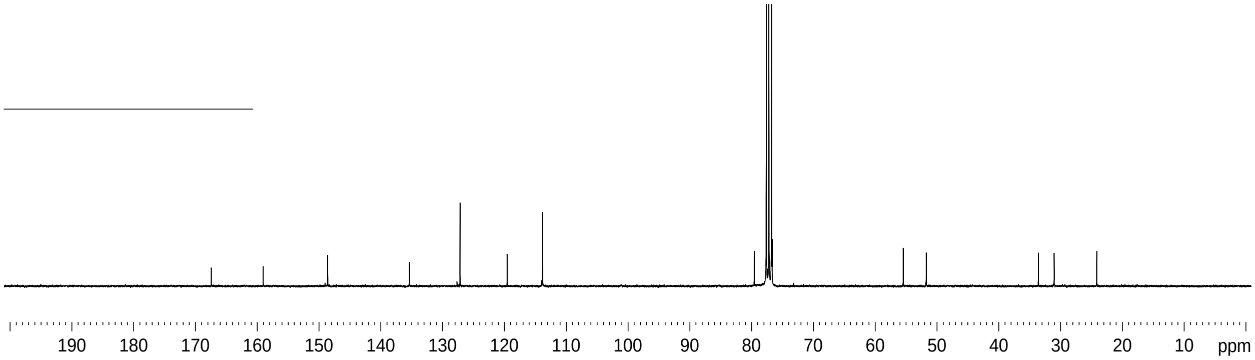

^{13}C NMR (expansion)

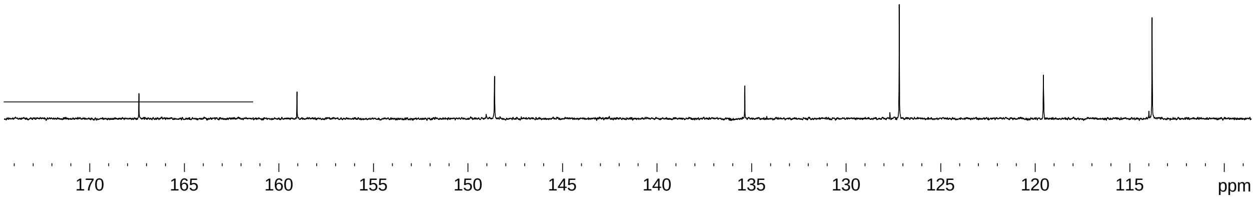

^{13}C NMR (expansion)

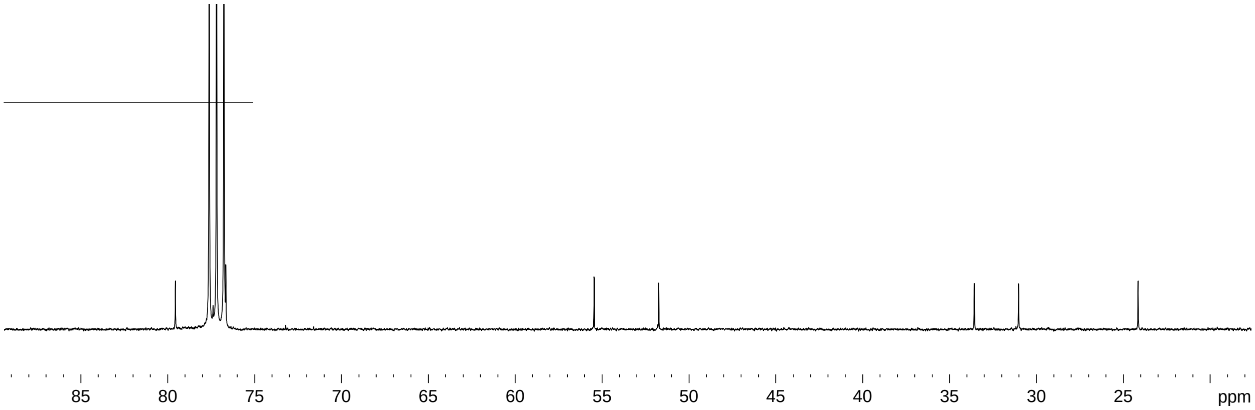

^{1}H and ^{13}C NMR spectra of 25

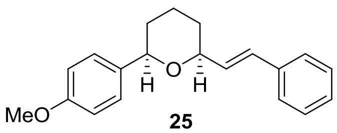

25

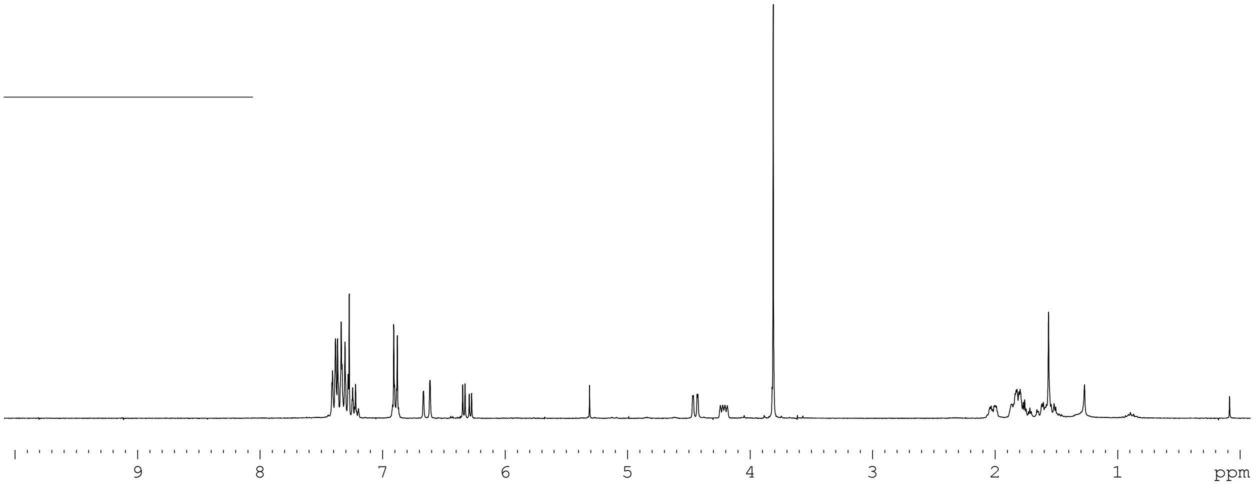

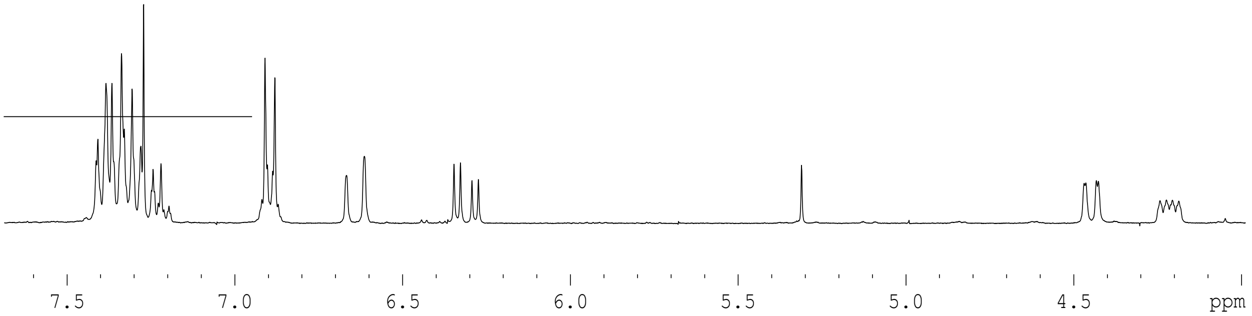

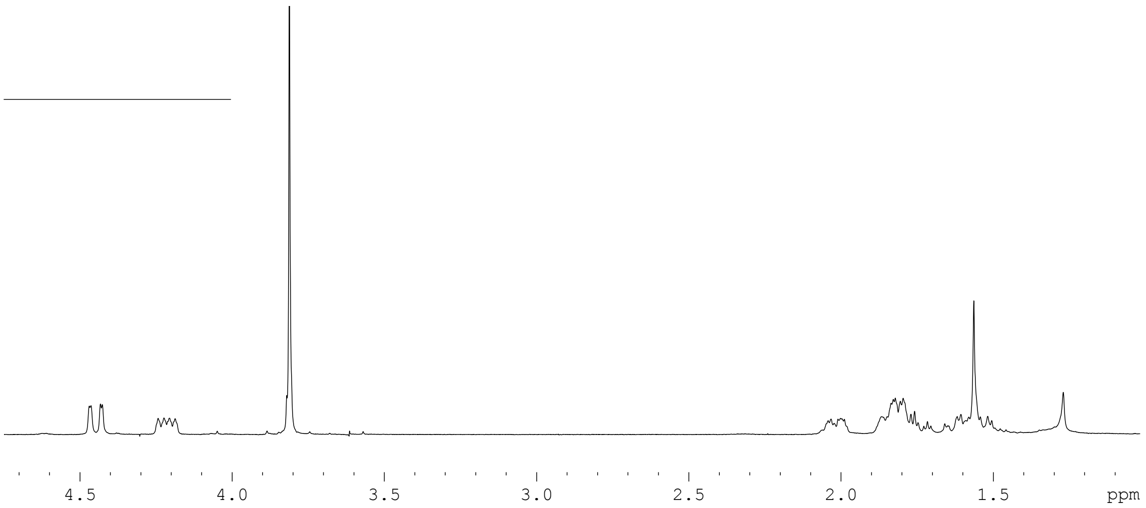

^{1}H and ^{13}C NMR spectra of 25 (cont)

^{13}C NMR (full)

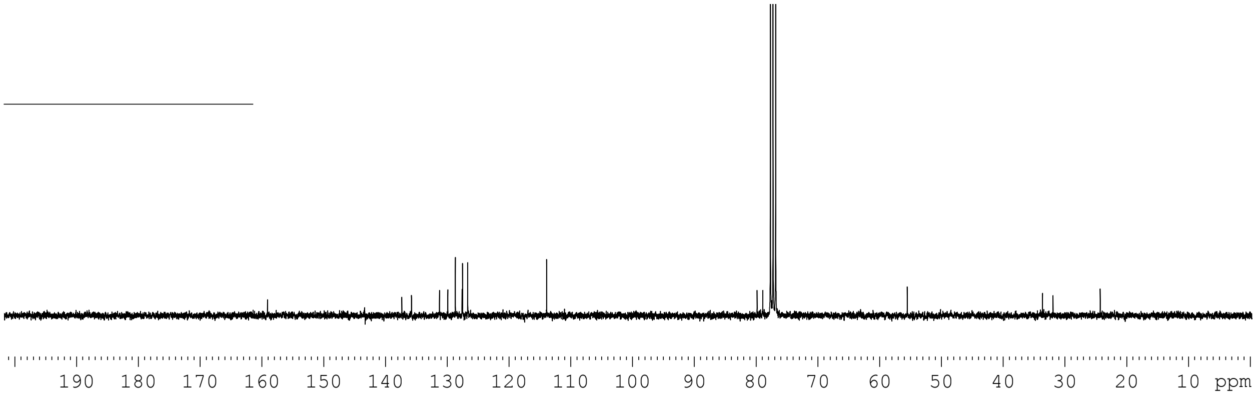

^{13}C NMR (expansion)

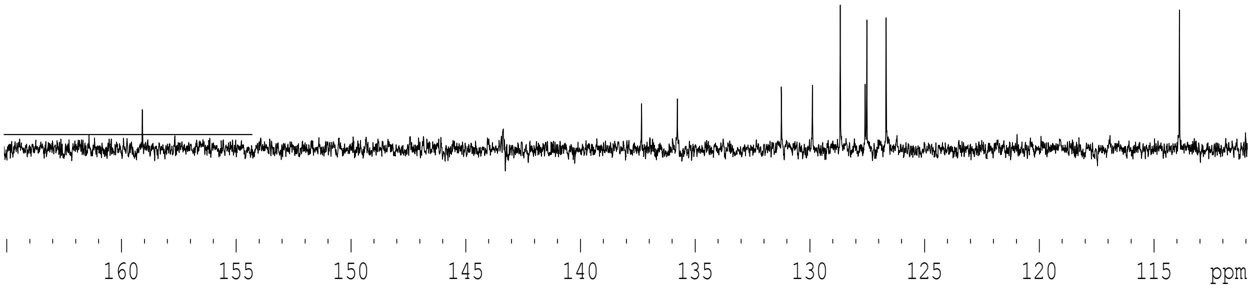

^{13}C NMR
(expansion)

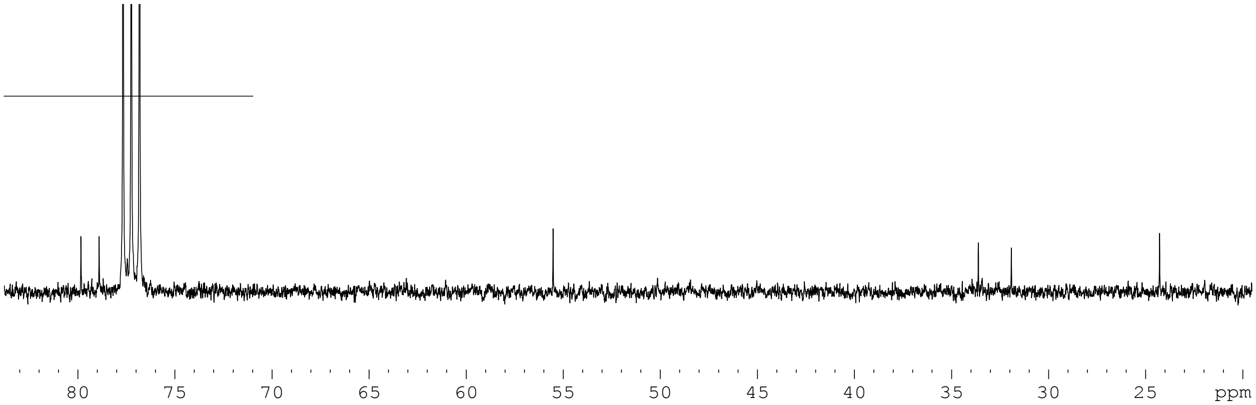

^{1}H and ^{13}C NMR spectra of 26

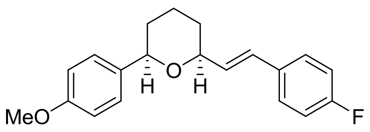

26

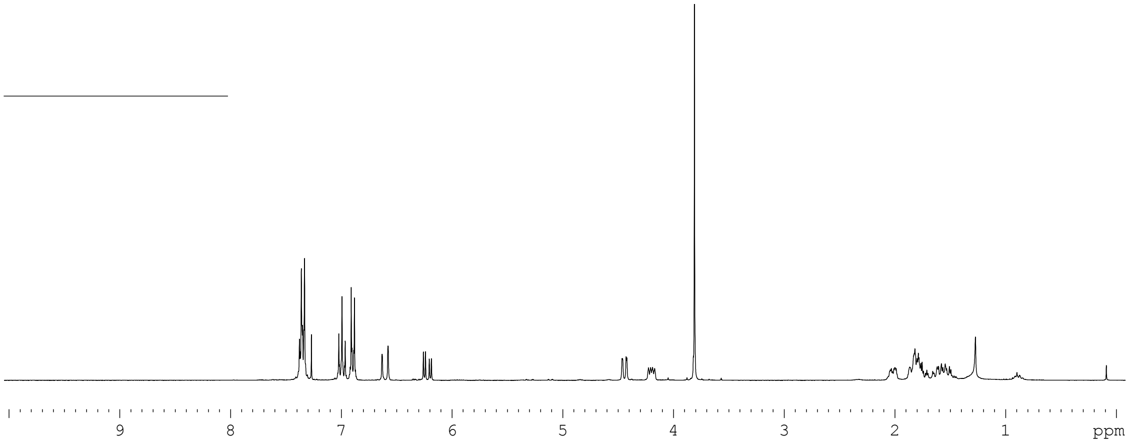

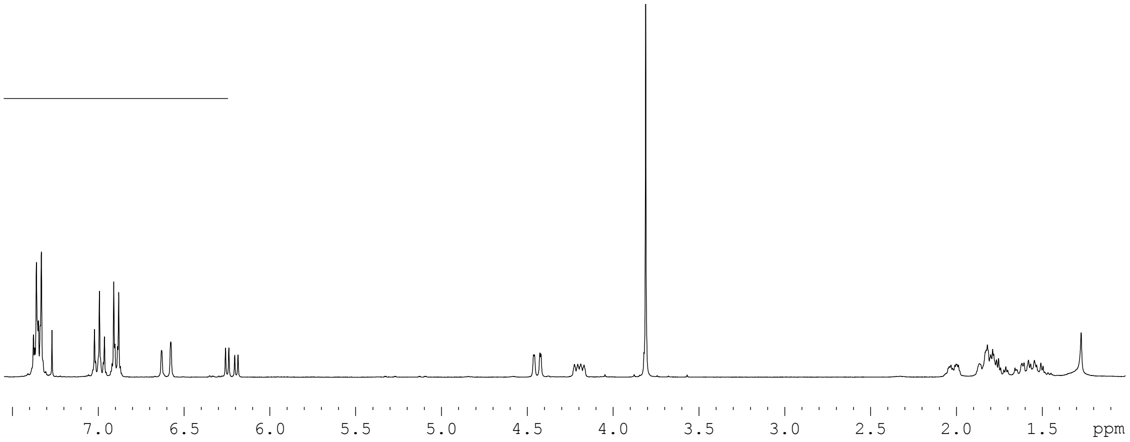

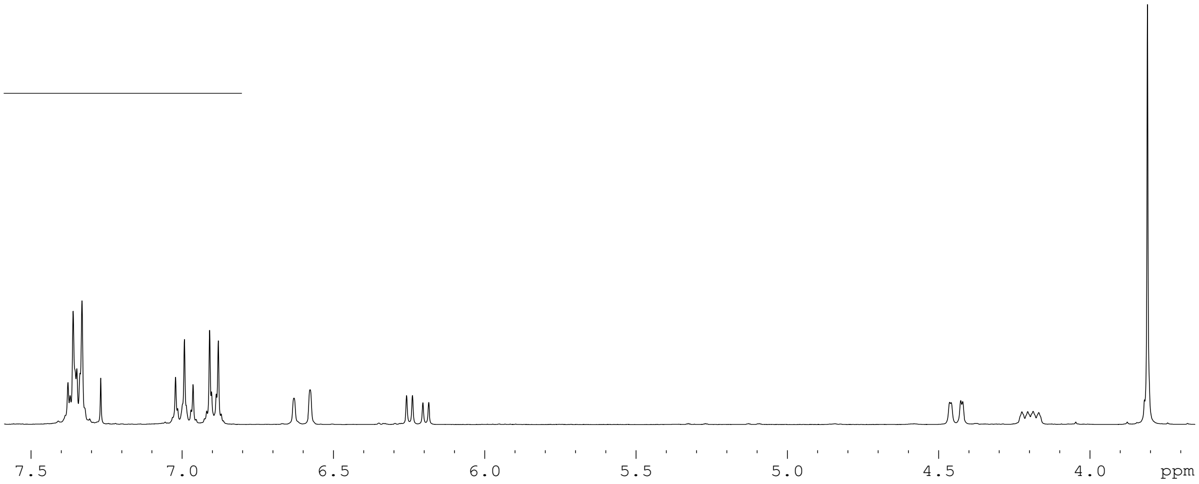

^{1}H and ^{13}C NMR spectra of 26 (cont)

^{13}C NMR (full)

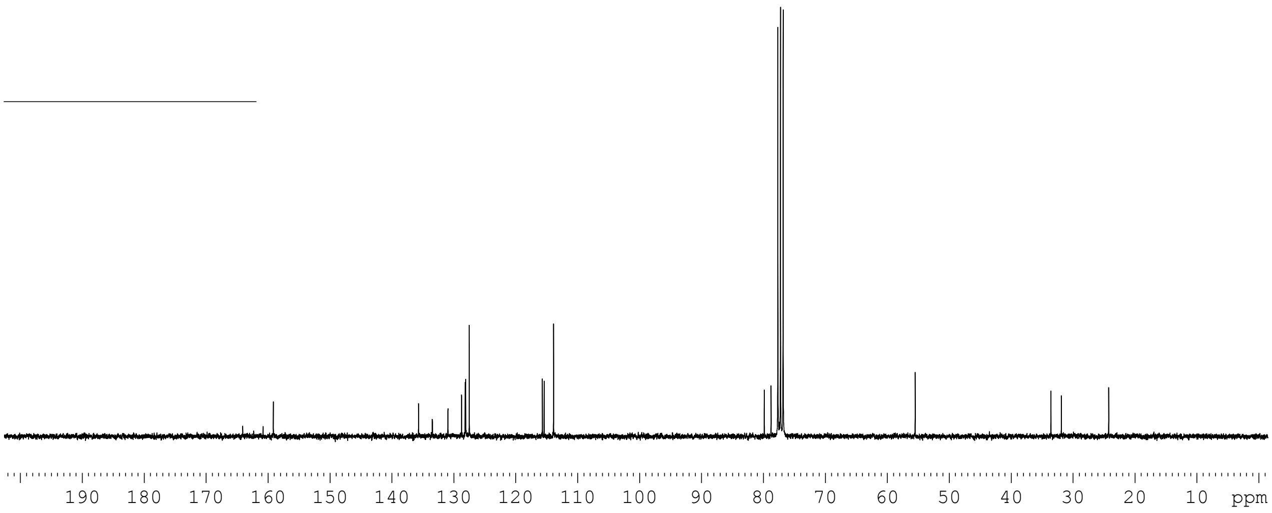

^{13}C NMR (expansion)

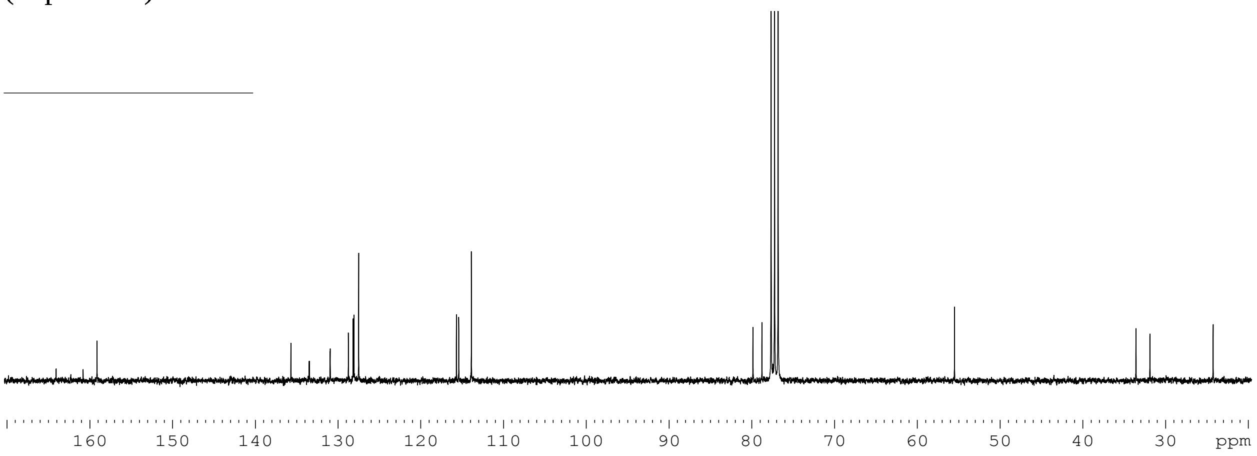

^{1}H-^{1}H COSY spectrum of 26

^{1}H-^{1}H COSY

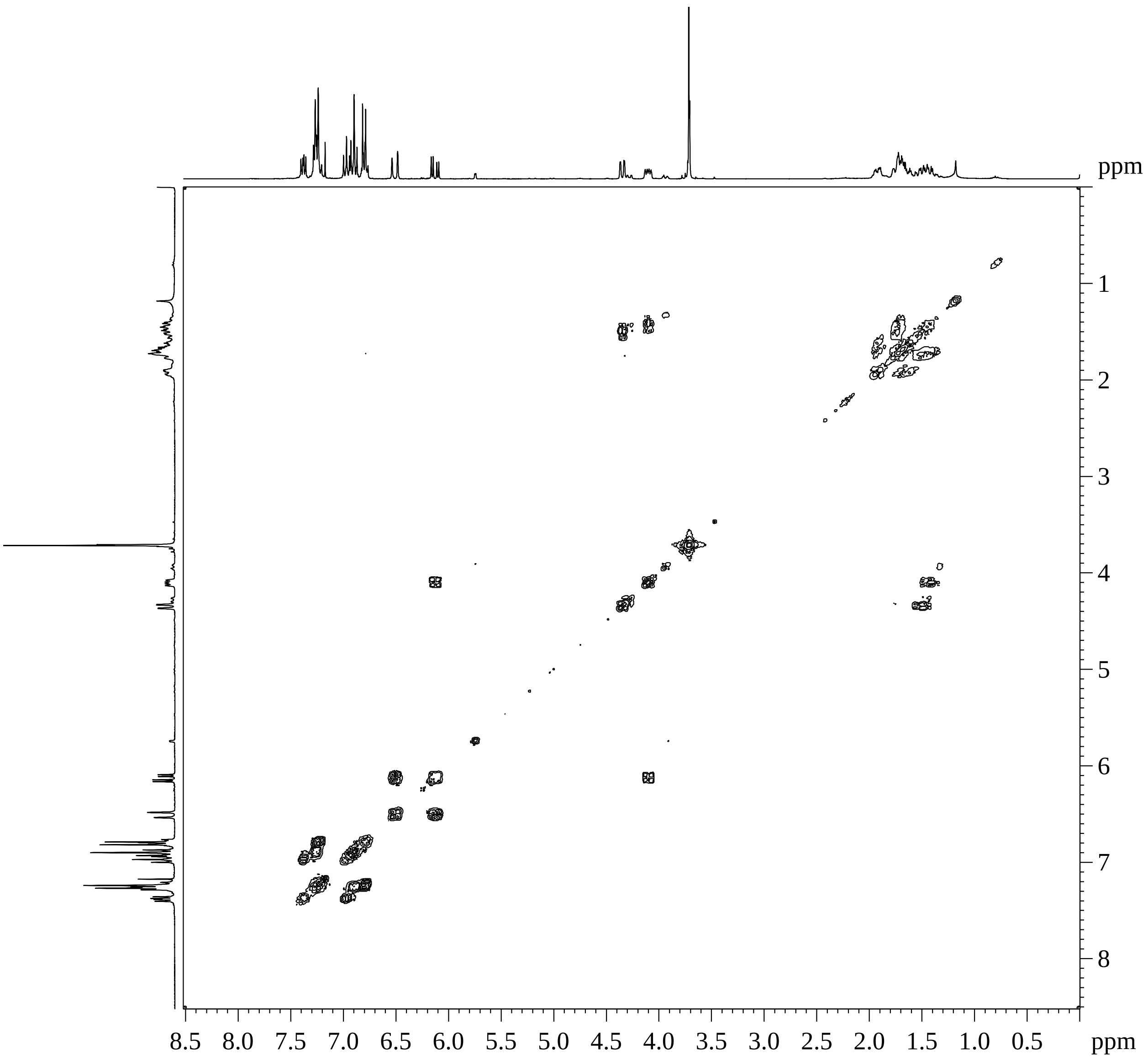

^{1}H NMR and ^{1}H-^{1}H COSY spectra of 7

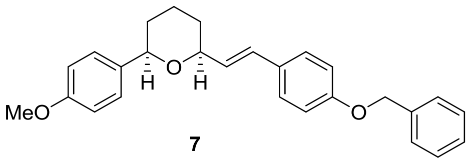

7

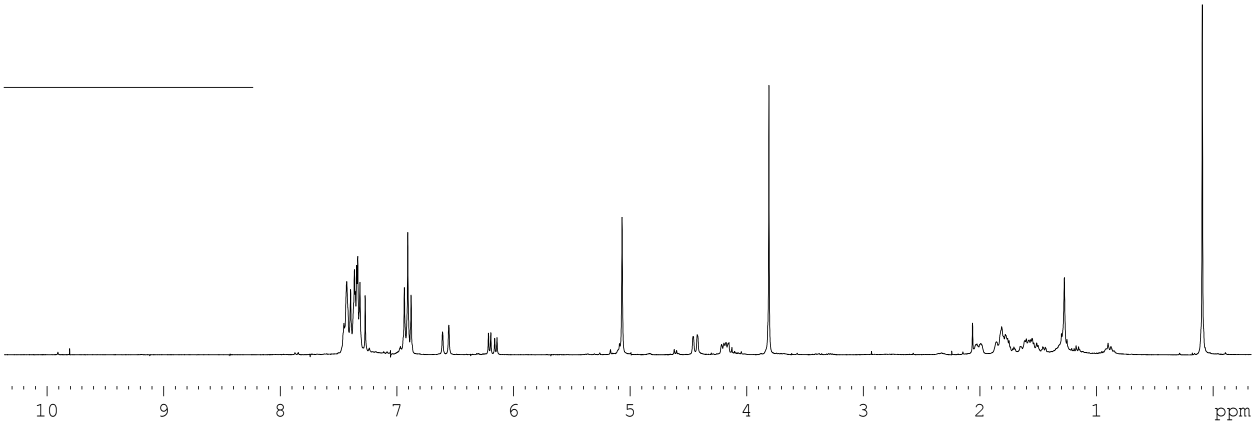

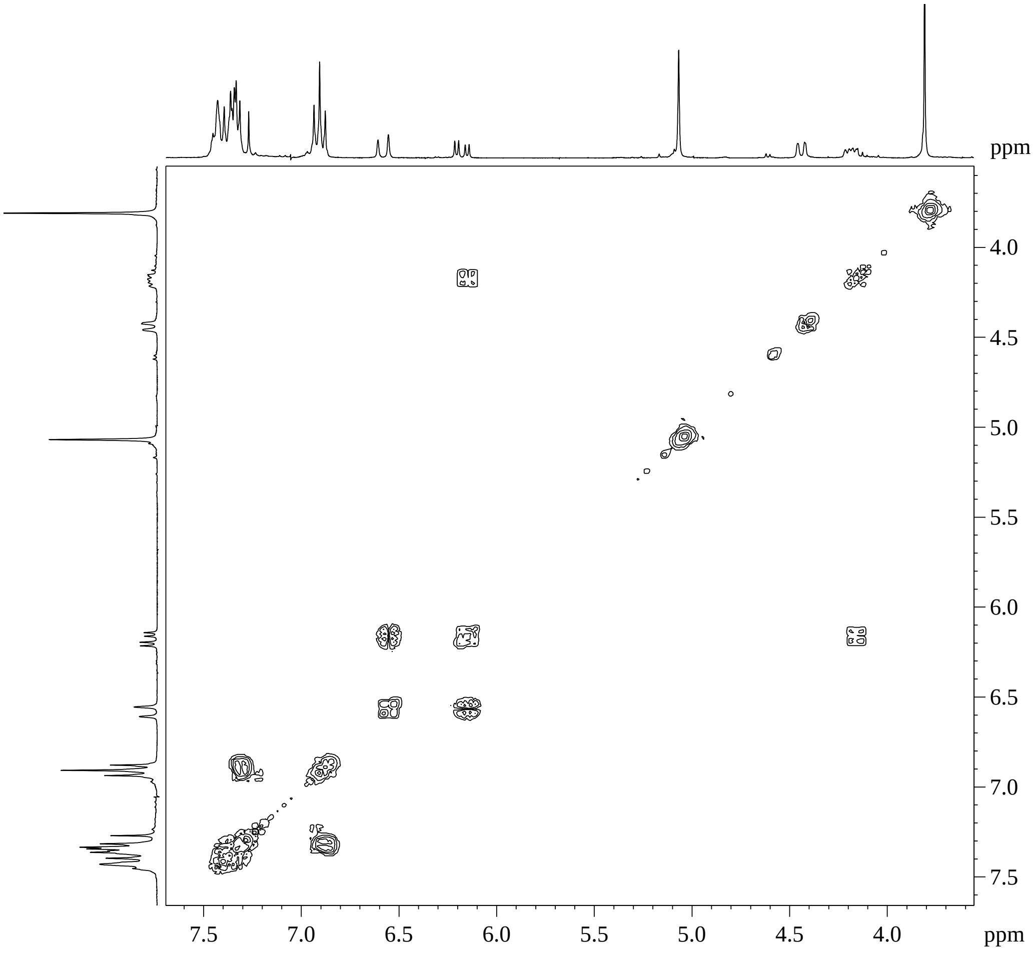

^{1}H NMR and ^{1}H-^{1}H COSY spectra of 7 (cont)

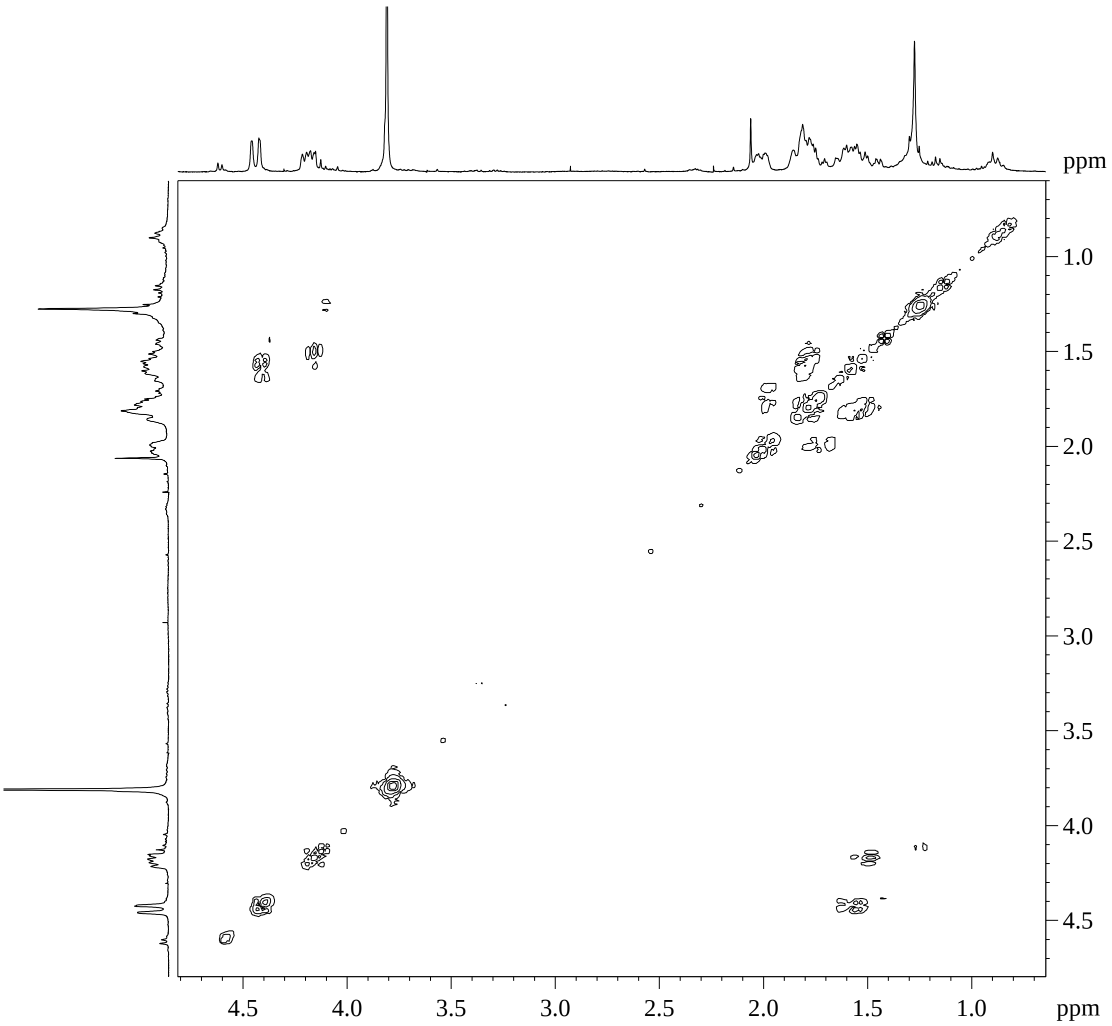

^{1}H and ^{13}C NMR spectra of 27

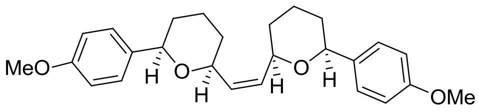

27

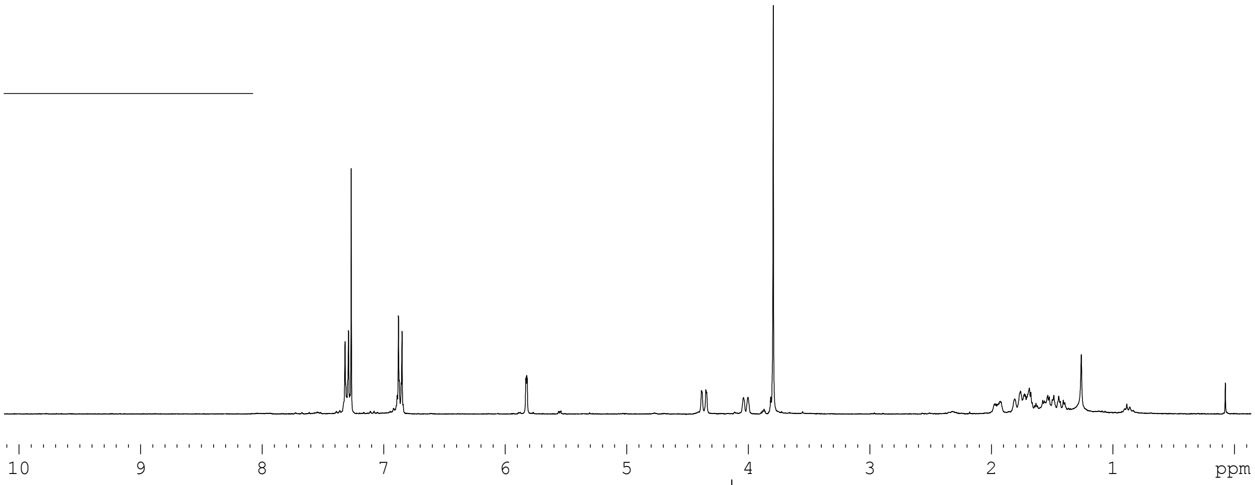

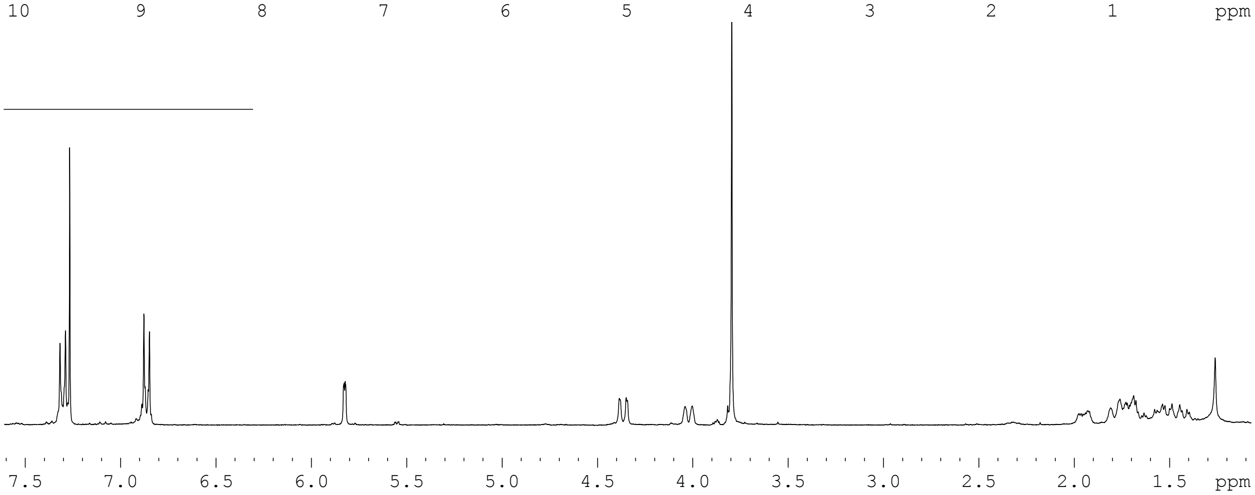

^{13}C NMR (full)

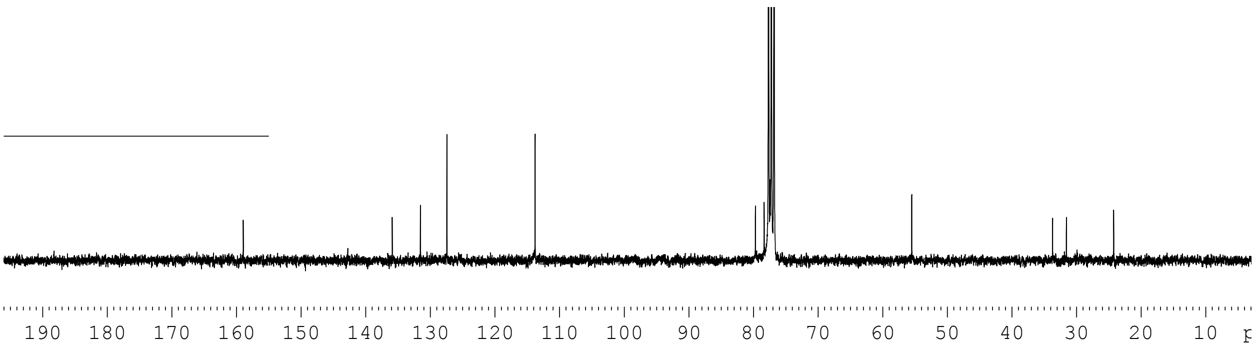

^{1}H-^{1}H COSY spectrum of 27

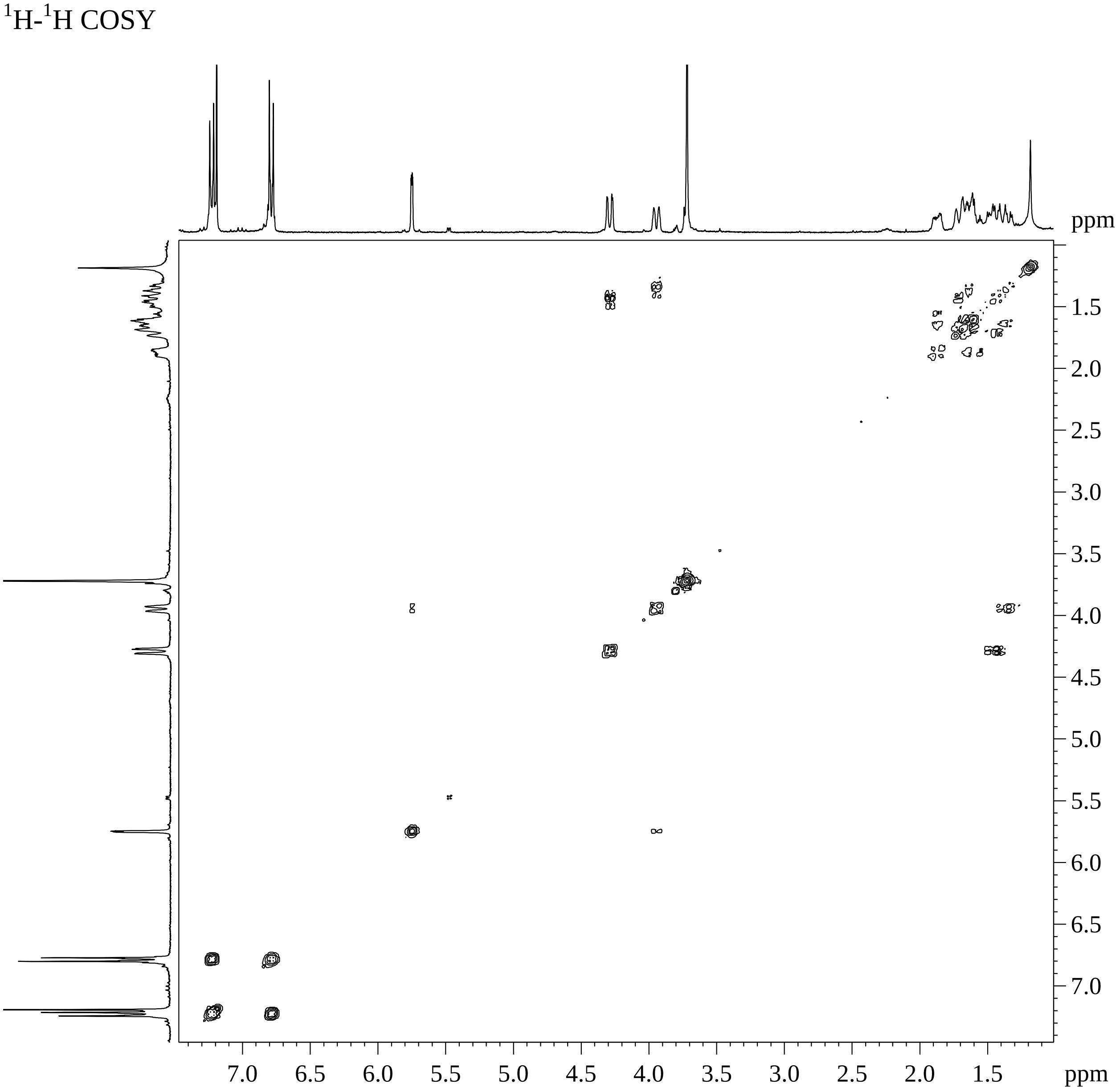

^{1}H, ^{31}P decoupled ^{1}H and ^{31}P NMR spectra of 22

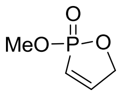

22

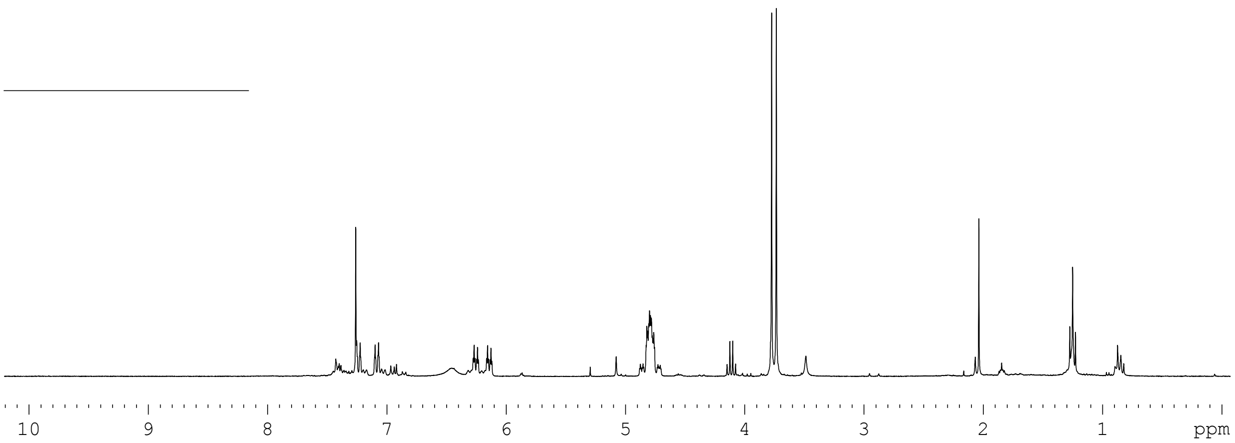

^{31}P decoupled ^{1}H NMR

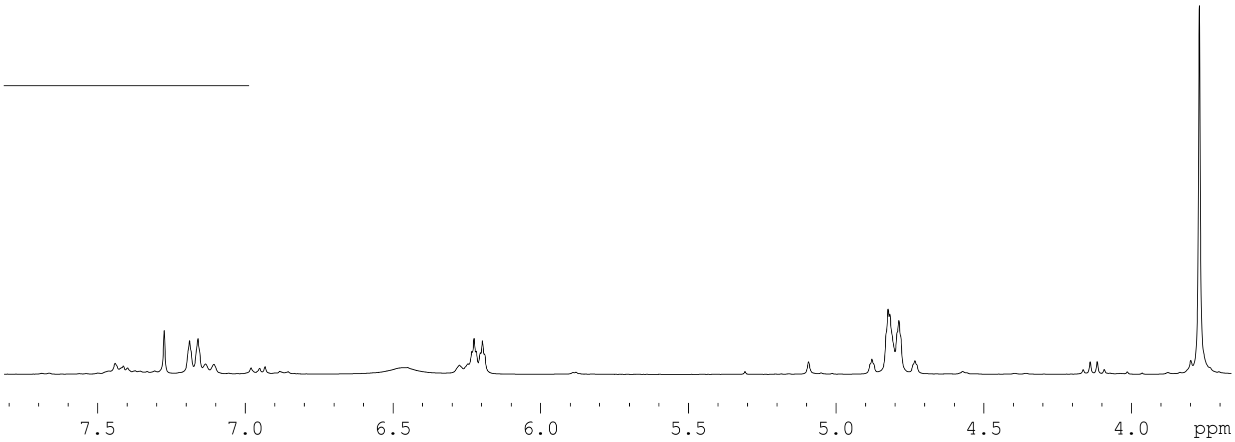

^{31}P NMR

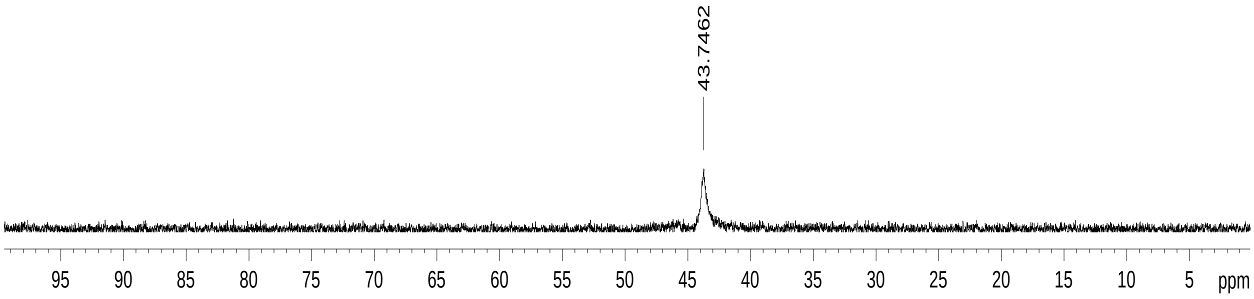

^{1}H-^{1}H COSY spectrum of 22

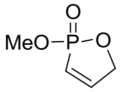

22

^{1}H-^{1}H COSY

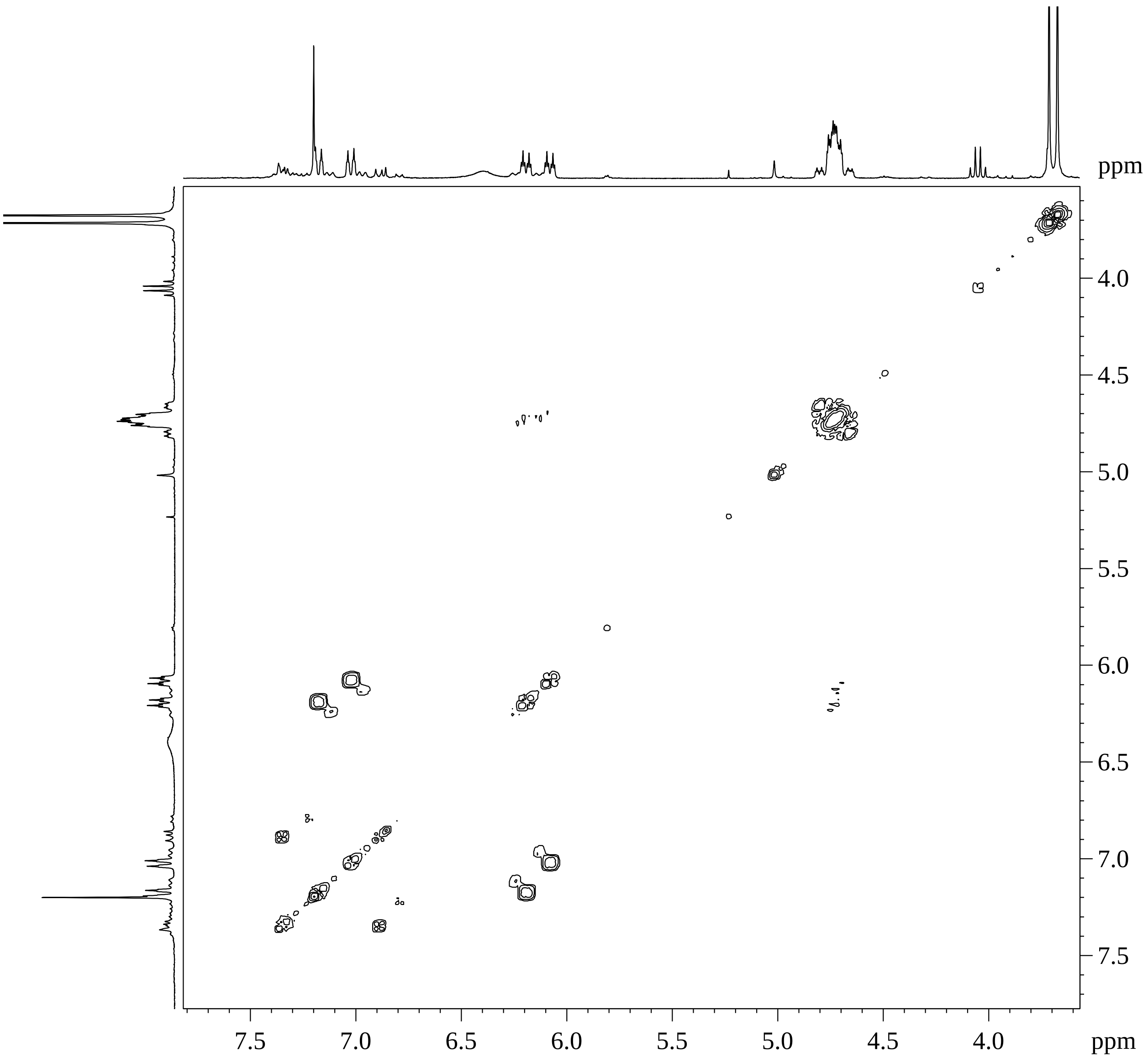

^{1}H and ^{13}C NMR spectra of 32

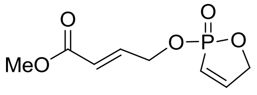

32

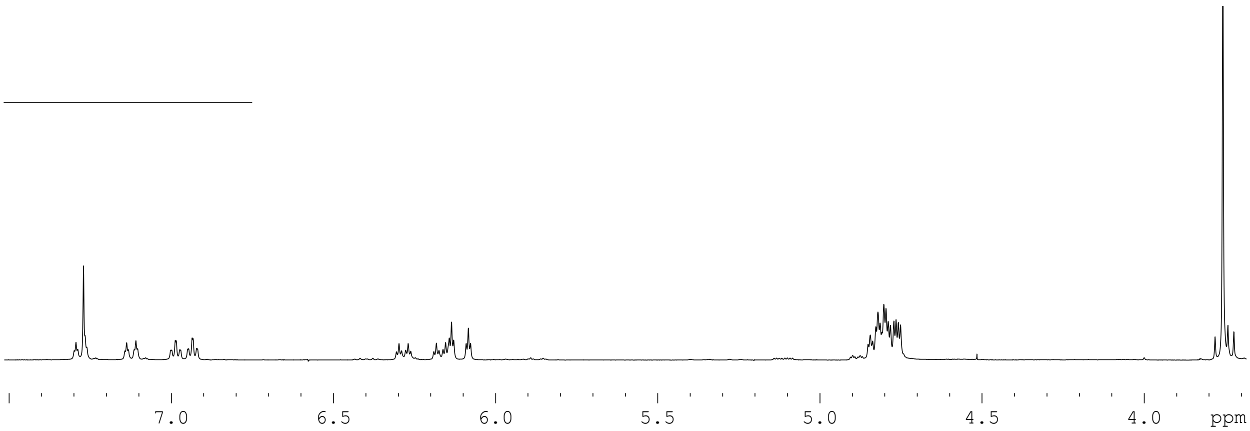

^{13}C NMR (full)

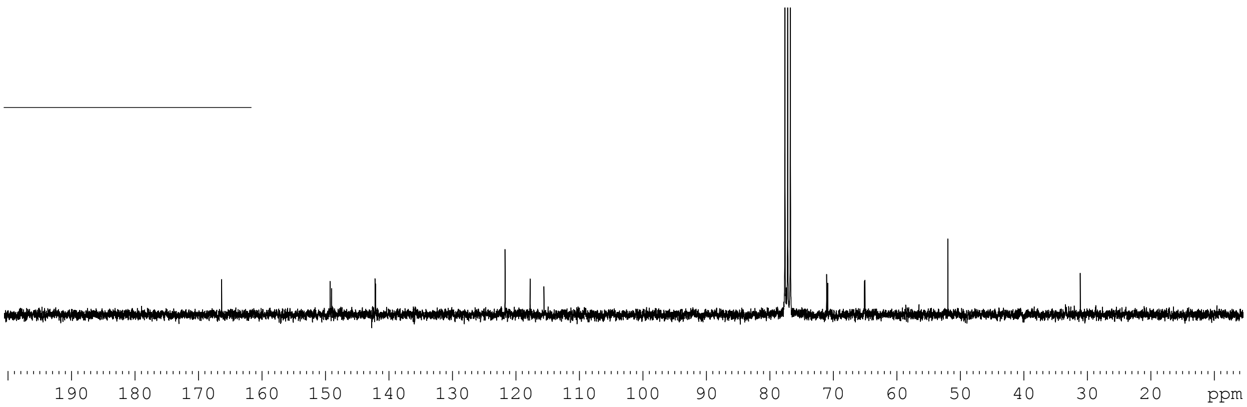

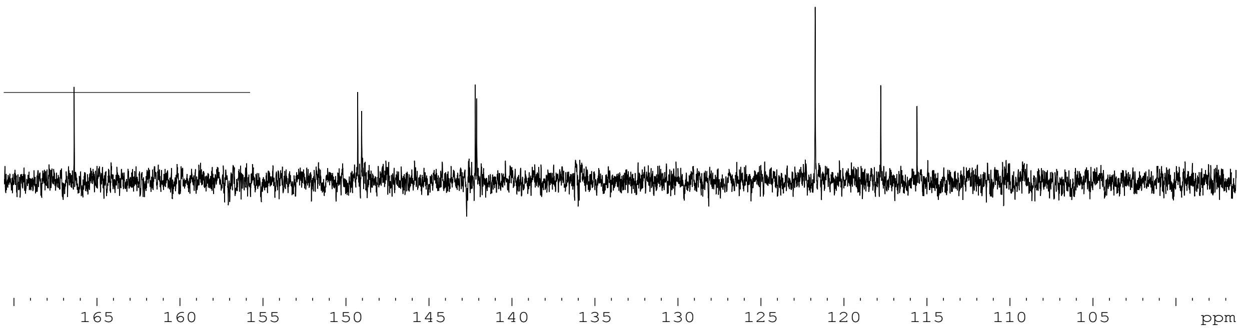

^{1}H-^{1}H COSY and ^{31}P NMR spectrum of 32

^{31}P NMR

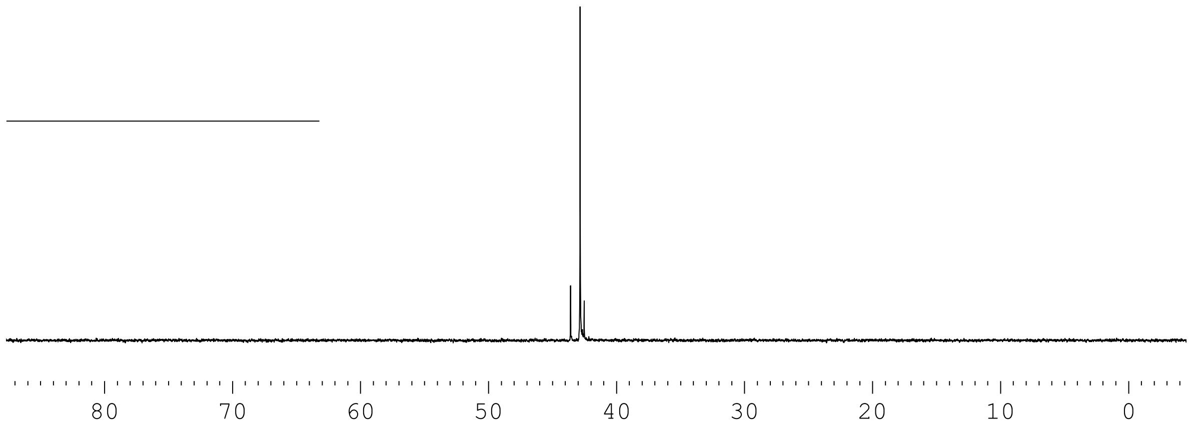

^{1}H-^{1}HCOSY

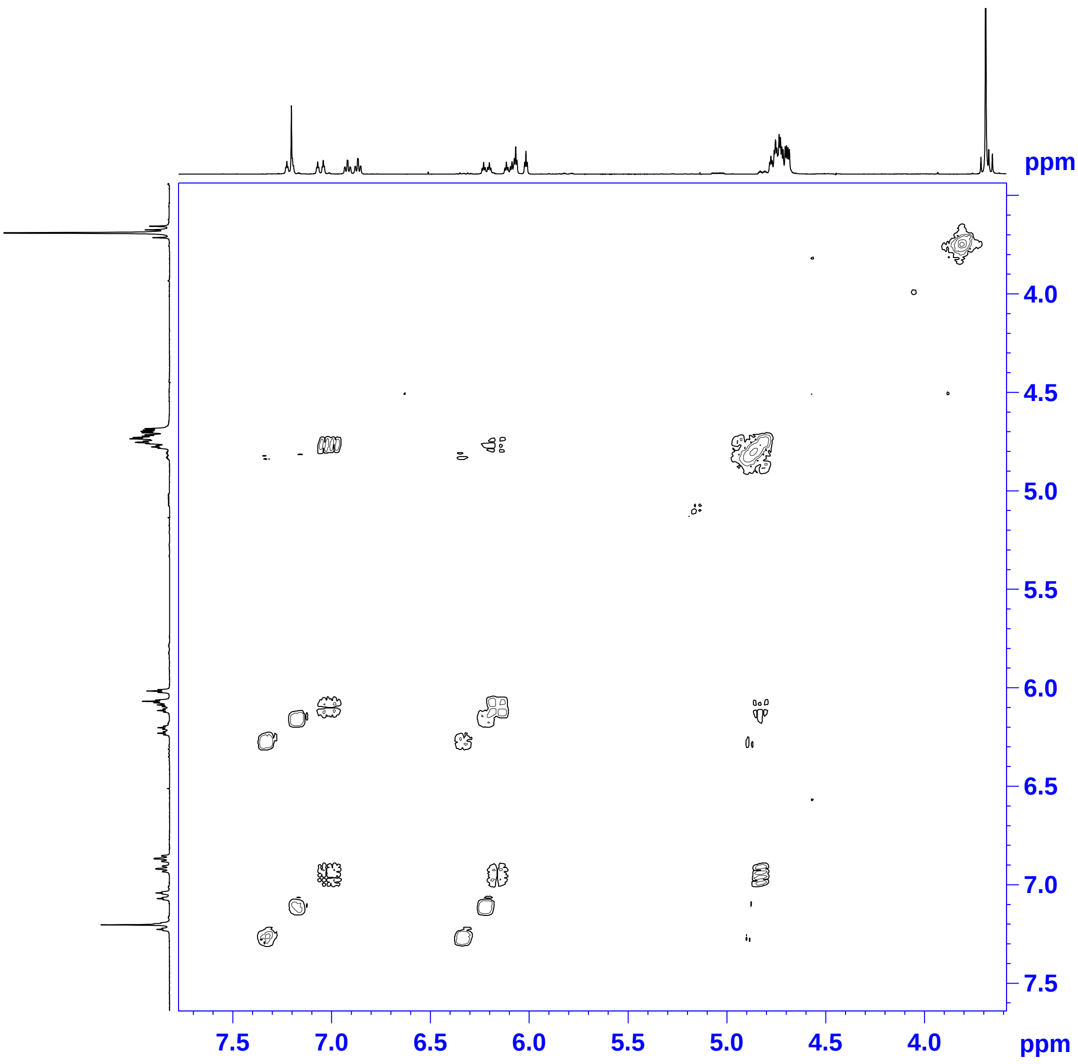

Cross metathesis of mixture of mono- and di-allyl vinyl phosphonates

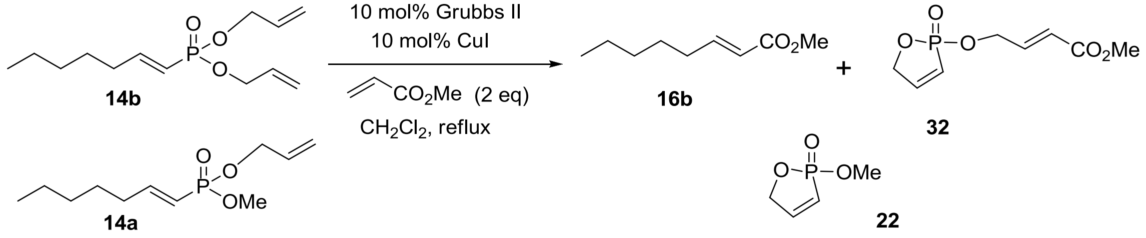

^{31}P NMR of mono- and di-allyl vinyl phosphonate (Reaction time, T = 0 min)

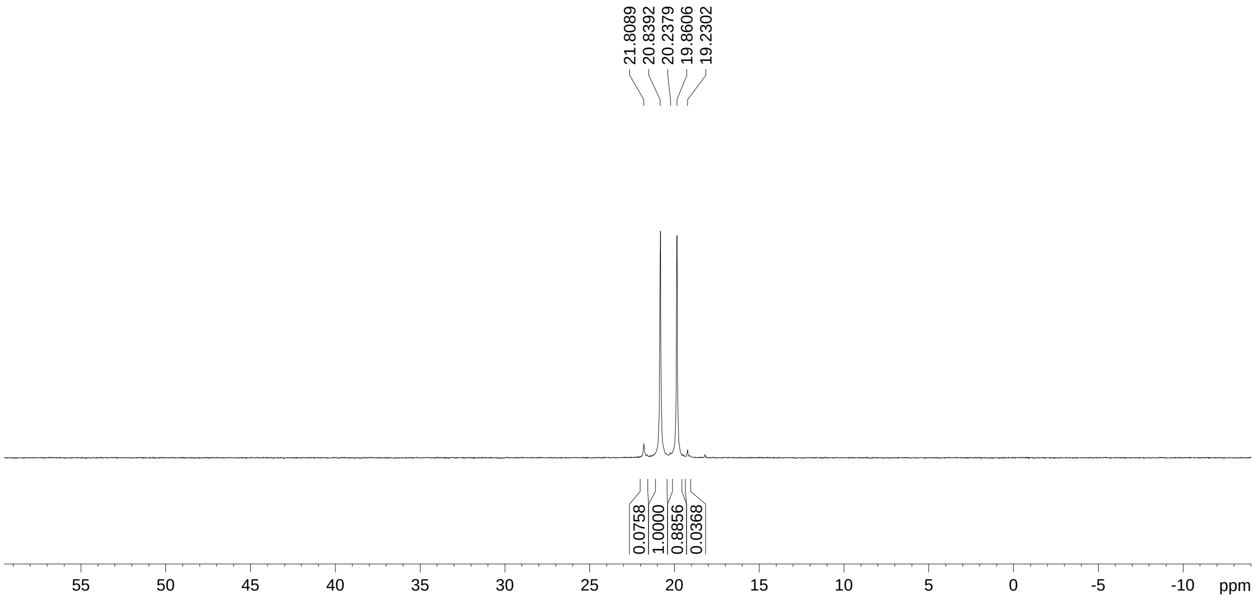

^{31}P NMR of reaction mixture

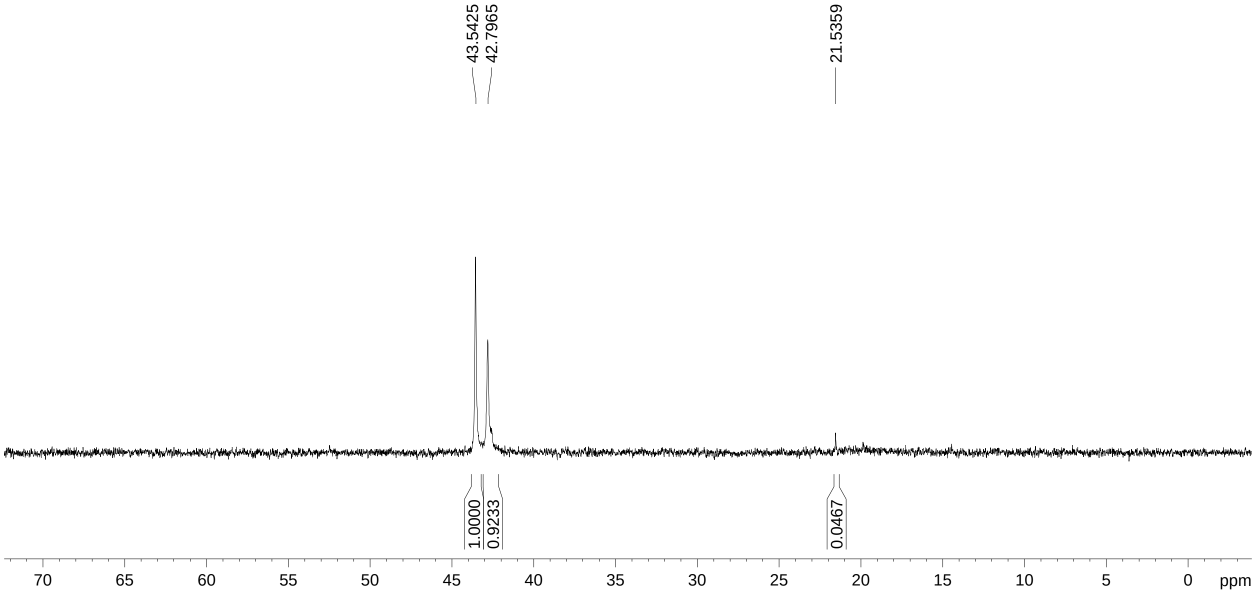

Supplement: File 1 — Experimental procedures, characterization data, 1H and 13C spectra for all new compounds. [file Beilstein_J_Org_Chem-10-1933-s001.pdf]
